# “Clickable” graphene nanoribbons for biosensor interfaces†

**DOI:** 10.1039/d3nh00590a

**Published:** 2024-02-22

**Authors:** Roger Hasler, Gonzalo E. Fenoy, Alicia Götz, Verónica Montes-García, Cataldo Valentini, Zijie Qiu, Christoph Kleber, Paolo Samorì, Klaus Müllen, Wolfgang Knoll

**Affiliations:** a AIT Austrian Institute of Technology GmbH 3430 Tulln Austria; b Laboratory for Life Sciences and Technology (LiST), Faculty of Medicine and Dentistry, Danube Private University 3500 Krems Austria wolfgang.knoll@dp-uni.ac.at roger.hasler@dp-uni.ac.at; c Instituto de Investigaciones Fisicoquímicas Teóricas y Aplicadas (INIFTA), Departamento de Química, Facultad de Ciencias Exactas, Universidad Nacional de La Plata La Plata B1904DPI Argentina; d Max Planck Institute for Polymer Research Ackermannweg 10 D-55128 Mainz Germany; e Université de Strasbourg, CNRS, Institut de Science et d’Ingénierie Supramoléculaires 8 allée Gaspard Monge 67000 Strasbourg France; f Centre for Advanced Technologies, Adam Mickiewicz University Uniwersytetu Poznańskiego 10 61-614 Poznań Poland; g School of Science and Engineering, Shenzhen Institute of Aggregate Science and Technology, The Chinese University of Hong Kong, Shenzhen (CUHK-Shenzhen) Guangdong 518172 P. R. China

## Abstract

We report on the synthesis of “clickable” graphene nanoribbons (GNRs) and their application as a versatile interface for electrochemical biosensors. GNRs are successfully deposited on gold-coated working electrodes and serve as a platform for the covalent anchoring of a bioreceptor (*i.e.*, a DNA aptamer), enabling selective and sensitive detection of Interleukin 6 (IL6). Moreover, when applied as the intermediate linker on reduced graphene oxide (rGO)-based field-effect transistors (FETs), the GNRs provide improved robustness compared to conventional aromatic bi-functional linker molecules. GNRs enable an orthogonal and covalent attachment of a recognition unit with a considerably higher probe density than previously established methods. Interestingly, we demonstrate that GNRs introduce photoluminescence (PL) when applied to rGO-based FETs, paving the way toward the simultaneous optical and electronic probing of the attached biointerface.

## Abstract 2

We demonstrate the synthesis and application of “clickable” graphene nanoribbons (GNRs) as a versatile interface for electrochemical biosensors.

## Graphical abstract

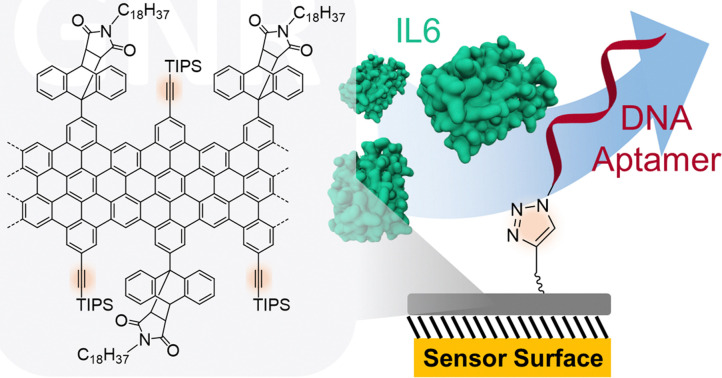

**New concepts**

In recent years, the development of novel two-dimensional materials, particularly graphene and its derivatives, has significantly advanced biosensor devices for healthcare applications. Electrochemical sensing, especially utilizing graphene-based technologies, emerges as a promising approach due to its cost-effectiveness, ease of operation, and compatibility with point-of-care devices. While functionalization strategies usually involve non-covalent methods to maintain the chemical structure and electrical properties of graphene-based materials, covalent strategies, *e.g.*, *via* click chemistry, result in a more robust attachment of the nanoscale biointerface. In this work we demonstrate for the first time the synthesis and use of “clickable” graphene nanoribbons (GNRs) as an intermediate linker for covalently anchoring a bioreceptor for a sensitive and selective analyte detection. More importantly, we show that GNRs introduce photoluminescence when applied to reduced graphene oxide-based field-effect transistors, allowing the concurrent optical and electronic probing of the attached biointerface. We believe that the use of “clickable” GNRs will open new avenues for these electronic devices for a broad range of applications in medical diagnostics and environmental health monitoring.

In recent years, the development of novel two-dimensional (2D) materials has played a pivotal role in advancing biosensor devices for healthcare applications due to their unique properties.^1^ These materials possess remarkable attributes such as a high surface-to-volume ratio, exceptional electrical conductivity, and biocompatibility. Additionally, their ultrathin nature allows efficient interaction with biomolecules, enhancing sensitivity in detecting various biological markers. The inherent properties of 2D materials facilitate the development of highly sensitive and selective biosensors, enabling accurate and rapid detection of biomarkers associated with health conditions, thereby contributing significantly to healthcare diagnostics and monitoring. Among the array of technologies proposed for healthcare applications, electrochemical sensing emerges as the most promising, primarily due to its cost-effectiveness, ease of operation, high sensitivity, and compatibility with point-of-care (PoC) devices.^2^ This technology has been adeptly integrated into wearable, portable, and implantable systems.^3^ For instance, electrochemical devices based on single-walled carbon nanotube screen-printed electrodes have been successfully employed for a single-step monitoring of the SARS-CoV-2 Spike Protein.^4^

Typically, a covalent attachment of the specific bio-receptor (*e.g.*, DNA/PNA capture probes, antibodies, nanobodies, and odorant binding proteins) on graphene-based surfaces is preferred in order to control the receptor's orientation, yielding a stable and robust sensor device.^5^ Unfortunately, an intermediary functionalization step becomes necessary due to the absence of suitable functional groups within the graphene derivatives for the direct anchoring of these bioreceptors. This initial functionalization process requires a molecule, namely an intermediate linker, possessing appropriate anchoring groups capable of binding to graphene, while also featuring reactive sites that can interact with the bioreceptor for effective attachment. Utilizing “click” chemistry as a potential strategy for covalently linking the recognition unit to the intermediate linker exhibits significant promise for applications in organic electronics.^6^ This approach comprises of highly reliable and efficient coupling reactions utilizing mild reaction conditions and accessible starting materials.^7^ Thereof, the Huisgen 1,3-dipolar cycloaddition of azides and alkynes is most frequently employed due to the bio-orthogonality and biocompatibility of the involved functional groups.^8^ Common intermediate linkers usually comprise aryl diazonium compounds containing an orthogonal functional group, *e.g.*, an alkyne modification.^9,10^ Unfortunately, the electrochemical grafting of diazonium compounds on graphene has an impact on its electronic properties, affecting the performance of the sensor device.^11^ Therefore, there is an urgent need for advanced covalent functionalization strategies of graphene or its derivatives without affecting their electrical properties. Such strategies would offer opportunities for improving sensitivity, selectivity, and biocompatibility, thereby augmenting the potential utility of graphene in various healthcare sectors, from diagnostics to therapeutics.

Among various graphene derivatives, graphene nanoribbons (GNRs) have garnered significant attention in biosensing due to their unique properties.^12^ GNRs are narrow strips of graphene with tuneable electronic and optical properties defined by their chemical structure, especially the width and edge configuration.^13,14^ Variations in width and edge structure can dictate the bandgap, electronic conductivity, and other properties critical for potential applications in nano- and optoelectronics and sensing *via* electrical or electrochemical readout.^15^ Additionally, GNRs are considered promising candidates for electrochemical sensing due to their excellent electron transport properties, high mechanical strength, large surface area, and, more importantly, ease of functionalization.

Numerous approaches have been reported for the synthesis of GNRs with atomic precision.^14^ Solution-based synthesis stands out due to its ability to easily introduce various functional groups onto the edges of GNRs.^14^ This synthetic process facilitates subsequent covalent anchoring of bioreceptor units and, simultaneously, prevents electronic alterations,^16^ enabling the integration of GNRs as interfaces in biosensing devices.^12^ While the concept of covalent and orthogonal functionalization of GNRs *via* click chemistry (*i.e.*, “clickable” GNRs) has been reported, their application in sensing remains unexplored.^17^

In this work, we combine the exceptional properties of GNRs with the “click” chemistry approach utilizing functionalized GNRs as biointerfaces in electrochemical sensors. We introduce a synthetic route for this material and show that GNR containing an alkyne-modification on their edges can be successfully deposited onto gold-coated working electrodes and post-functionalized with a bioreceptor (*i.e.*, DNA aptamer modified with an azide-group) for the selective and sensitive detection of IL6, an inflammatory response protein. Additionally, we explore the unique advantages offered by a “clickable” GNR interface when applied as an intermediate linker on rGO based field-effect transistors (FETs) for label-free, multivariable detection of analytes.

## Material and methods

### Materials

Tetrabutylammonium fluoride (TBAF, 1 M in THF), dimethyl sulfoxide (DMSO), copper sulfate (CuSO_4_), sodium ascorbate (NaAsc), ethylenediaminetetraacetic acid (EDTA), bovine serum albumin (BSA, lyophilized powder, ≥96%), (3-aminopropyl)triethoxysilane (APTES, 99%), hydrazine monohydrate (64–65%, reagent grade 98%), *n*-octadecanthiol (*n*ODT), and Hellmanex^TM^III solution were purchased from Sigma Aldrich. Potassium hexacyanoferrate(ii) trihydrate (K_4_[Fe(CN)_6_]·3H_2_O) was purchased from Merck. Phosphate Buffered Saline (PBS) tablets, ethanol (EtOH, absolute, 99.8%), and tetrahydrofuran (THF) were purchased from VWR. Azido-ferrocene (N_3_-Fc) was obtained from Baseclick GmbH. Graphene oxide water dispersion (0.4 wt%) was obtained from Graphenea. The azide-modified IL6 DNA aptamer (/5AzideN/TT TTT TCT TCC AAC GCT CGT ATT GTC AGT CTT TAG T, ∼11 kDa) was custom synthesized by Integrated DNA Technologies. Recombinant Human Interleukin-6 (IL6, 21.1 kDa) was purchased from ReliaTech GmbH. All solutions were prepared with MilliQ-grade DI-H_2_O. All chemicals were purchased from commercial sources and used without further purification unless otherwise noted. All reactions dealing with air- or moisture-sensitive compounds were carried out in a dry reaction vessel under argon.

1,3-Bis(4-iodophenyl)propan-2-one was prepared according to reported procedures.^18^

### Characterization of GNRs

Preparative column chromatography was conducted with silica gel from Macherey Nagel with a grain size of 0.063–0.200 mm or 0.04–0.063 mm. Analytical thin layer chromatography (TLC) was performed on silica gel coated substrates Alugram Sil G/UV254.

Solution nuclear magnetic resonance (NMR) spectra were recorded on Bruker AVANCE 300 MHz and 700 MHz spectrometers, and referenced to residual signals of the deuterated solvent. Chemical shifts were reported in ppm. Abbreviations: s = singlet, d = doublet, t = triplet, m = multiplet. High-resolution mass spectrometry (HRMS) was taken on a SYNAPT G2 Si high-resolution time-of-flight (TOF) mass spectrometer (Waters Corp., Manchester, UK) by matrix-assisted laser desorption/ionization (MALDI). MALDI-TOF MS analysis of polyphenylene precursor 6 and 8 was performed using trans-2-[3-(4-tert-butylphenyl)-2-methyl-2-propenylidene]malononitrile (DCTB) as matrix.

Analytical size exclusion chromatography (SEC) was performed on SDV PSS GPC columns using tetrahydrofuran (THF) as eluent at a temperature of 303 K. Absorbance was determined on a UV S-3702 detector (SOMA) at a fixed wavelength of 270 nm. The samples were referenced with respect to standard polystyrene (PS) as well as poly(*para*-phenylene) (PPP) calibration curves.

### Fabrication of GNR-functionalized electrodes

Working electrodes utilized in this study consist of gold-coated glass substrates fabricated in-house. Briefly, glass slides (3 × 3 cm) were cut from BK7 glass substrates and cleaned by subsequent sonication for 15 minutes in Hellmanex^TM^ III (1% v/v in MilliQ-grade DI-H_2_O), MilliQ-grade DI-H_2_O, and EtOH. Clean substrates were dried under compressed air, loaded into a thermal evaporator (Auto306 Lab Coater from HHV Ltd), and coated with a 2 nm Cr followed by a 50 nm Au layer. As-prepared substrates were stored under Argon until further usage.

Electrode substrates were cleaned by sonication for 1 hour in EtOH and dried under a stream of N_2_ prior to the formation of the self-assembly monolayer (SAM), adapted from previously reported procedures.^19,20^ The formation of a *n*ODT SAM layer was achieved by incubating the substrates for 1 hour in a 1 mM *n*ODT solution in EtOH, after which the substrates were rinsed with EtOH and dried under a stream of N_2_.^21^ SAM-functionalized substrates were pre-heated to 100 °C on a hot plate. While still on the hot plate, 100 μL of the GNR solution in THF (1 mg mL^−1^, sonicated in a bath sonicator for 60 minutes) was drop-casted on top. After complete solvent evaporation, the substrates were removed from the hot plate and cooled down to room temperature (RT).^22^

### Click-functionalization of GNRs

The procedure for clicking an azide-modified probe to the alkyne-functionalized GNRs was adapted from a previously reported protocol.^6^ First, the TIPS-protected alkyne groups on the edges of the GNRs were deprotected using 0.1 M TBAF in THF for 1 hour and rinsed with THF. Subsequently, 50 μL of a 10 μM N_3_-Fc solution in DMSO was added to the desired area. To catalyze the click-reaction, 20 μL of a 1 : 1 mixture of CuSO_4_ (0.01 M in MilliQ-grade DI-H_2_O) and NaAsc (0.05 M in MilliQ-grade DI-H_2_O) was added. After 1 hour, the electrodes were rinsed with DMSO and MilliQ-grade DI-H_2_O. For the application as the biosensor, an IL6-DNA aptamer was clicked following the same approach by adding 10 μM of it in PBS 1X to the deprotected GNR-functionalized electrode substrate. Rinsing with DMSO was omitted in case of the DNA aptamer and remaining excess was removed by rinsing with MilliQ-grade DI-H_2_O. Any remaining copper catalyst was removed by incubating the substrates for 10 minutes in a 10 mM EDTA solution in MilliQ-grade DI-H_2_O, followed by rinsing with MilliQ-grade DI-H_2_O.

### Electrochemical characterization

Electrochemical measurements were carried out using an Autolab potentiostat. The electrochemical cell consisted of three electrodes in a Teflon-lined cell with 2 mL volume capacity. An Ag/AgCl reference electrode (RE) and a Pt wire as the counter electrode (CE) were utilized for all experiments. Cyclic voltammetry (CV) measurements were conducted in PBS 1X as the electrolyte.

### Electrochemical sensing of IL6

The sensing experiments were carried out by exposing the sensor surface to increasing concentrations of analyte in PBS 1X for 20 minutes, followed by rinsing the surface with PBS 0.1X. After each concentration, differential pulse voltammetry (DPV) and electrochemical impedance spectroscopy (EIS) measurements were performed in PBS 0.1X containing 5 mM [Fe(CN)_6_]^4−^.

### Fabrication of GNR-functionalized FETs

The fabrication of the FETs was adapted from a previously reported procedure.^23^ Briefly, drain-source substrates (channel length = 5 μm, Micrux Technologies Inc.) were functionalized with APTES by immersion in a 1% APTES solution in EtOH at RT for 1 hour. Subsequently, the substrates were rinsed with EtOH, dried under a stream of N_2_, and incubated at 120 °C for 1 hour. GO (1 : 5 dilution in MilliQ-grade DI-H_2_O) was centrifuged (60 seconds at 10 000 rpm) and the resulting supernatant was deposited onto the APTES-functionalized substrates *via* spin coating (1800 rpm, 60 seconds). GO-coated substrates were reduced in hydrazine vapour at 80 °C for 16 hours, followed by annealing at 200 °C under vacuum for 2 hours. GNRs were deposited onto as-prepared rGO-based FET substrates and further click-functionalized with the DNA aptamer following the above-mentioned procedures.

### Field-effect transistor characterization

FET measurements were carried out with a Keithley 4200-SCS probe station. An Ag/AgCl reference electrode was employed as the gate electrode. For all *I*_DS_*V*_G_ transfer measurements, a constant drain-source voltage (*V*_DS_) of 50 mV was applied. The gate voltage (*V*_G_) was swept between −500 and 500 mV. All experiments were performed using PBS 0.1X as the electrolyte.

### Spectroscopic and morphological characterization

Raman spectra were acquired with a Renishaw InVia Reflex system. The spectrograph used a high-resolution grating (2400 grooves cm^−1^) with additional bandpass filter optics, a confocal microscope, and a 2D-CCD camera. The excitation was performed using a 532 nm laser excitation beam, with a 100× objective, 0.2 mW maximum power, and 1 s acquisition time. The Raman spectra were processed using WiRE software V4.4 (Renishaw, U.K.). An XPS (Thermo Scientific K-Alpha X-ray photoelectron spectrometer) equipped with an aluminium X-ray source (energy 1.4866 keV) at a vacuum level of 10^−8^–10^−9^ mbar in the main chamber was used. The spot size of the X-ray beam was fixed at 400 μm. The XPS spectra were processed by using Avantage software. Scanning Electron Microscopy (SEM) images were recorded with a Zeiss SUPRATM 40 Field Emission Scanning Electron Microscope and a FEI Quanta FEG 250 instrument S3 (FEI corporate, Hillsboro, Oregon, USA).

## Results and discussion

Here we describe an efficient solution synthesis of particularly high soluble GNRs due to solubility-promoting AOM (anthryl- based *N-n*-octadecyl maleimide) groups in the periphery. The GNRs can undergo functionalization with TIPS-ethylene, setting the stage for subsequent Click reactions.

**Scheme 1:**
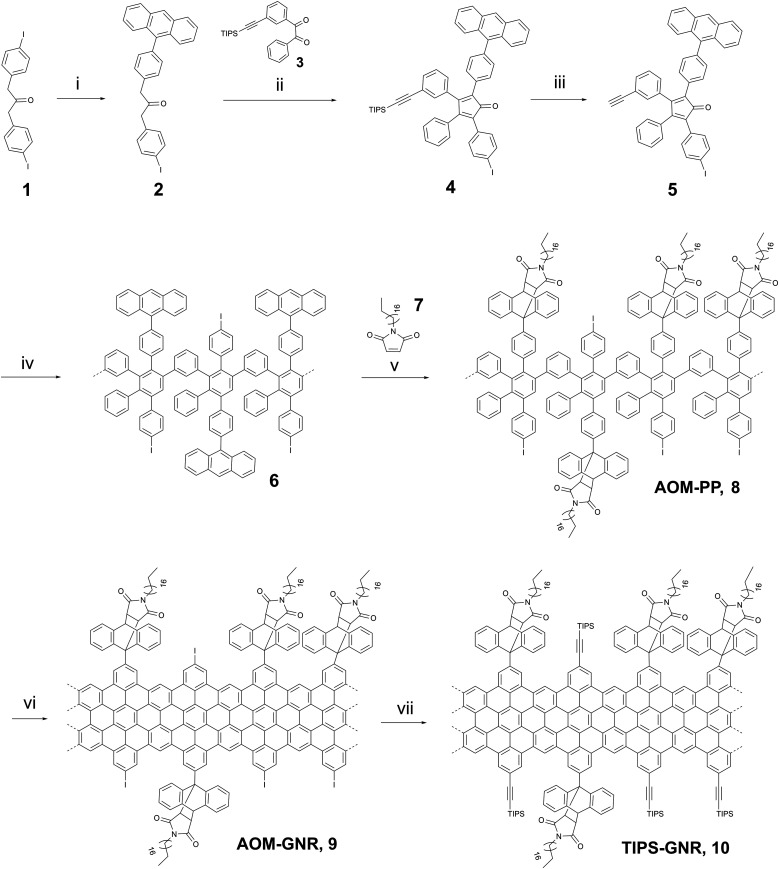
Synthetic route of “clickable” GNRs 10: (i) anthracen-9-yl-boronic acid, Pd(PPh_3_)_4_, K_2_CO_3_, THF, 12 h, 60 °C, 47%, (ii) NBu_4_NOH, 1,5 h, 65 °C, *t*-BuOH, 79%, (iii) TBAF, RT, CH_2_Cl_2_, 20 min, 67%, (iv) Ph_2_O, 250 °C, 36 h, 79%, (v) *o*-Xylene, 36 h, 150 °C, 92%. (vi) FeCl_3_ (2.76 × 10^−3^ M), CH_2_Cl_2_/MeNO_2_, 72 h, RT, 92%, (vii) ethynyltriisopropylsilane, THF/NEt_3_, 72 h, RT, 86%.

As demonstrated in Scheme 1 the synthetic route starts with the Suzuki–Miyaura cross-coupling between the diiodoacetone 1 and anthracene-9-yl-boronic acid to obtain compound 2 in a yield of 47%. The subsequent Knoevenagel condensation between compounds 2 and 3 provided the TIPS-protected cyclopentadienone 4 in a yield of 79%. Finally, the desired monomer 5 was produced by cleavage of the TIPS protecting group using TBAF with a yield of 67%. The structure of 5 was confirmed by NMR and high-resolution MALDI-TOF MS spectroscopy (Fig. S1, ESI†). The ^1^H NMR spectrum displayed the proton signal characteristic of terminal alkynes at a chemical shift of 3.10 ppm, indicating that the successfully deprotected cyclopentadienone 5 was obtained (see (ESI†)).

The obtained monomer 5 was subsequently reacted in a Diels–Alder polymerization in diphenyl ether at 250 °C for 36 hours to form the anthracene-containing polymer structure 6. The crude polymer was washed with methanol and fractionated *via* consecutive Soxhlet extraction using various solvents (methanol, acetone, ethyl acetate, and THF). However, the low molecular weight oligomers could not be completely separated, and the mixture was used in the next step without further purification. The polymer 6 and *N*-octadecylmaleimide 7 were ultimately converted into the desired target polymer AOM-PP8 by Diels–Alder cycloaddition, followed by examination through analytical GPC analysis (Fig. S2, ESI†).

Apart from the primary peak at approximately 14–24 minutes, the GPC profile exhibited two additional peaks at 28 and 32 minutes (Fig. S2a, ESI†). The peak at 28 minutes could be attributed to low molecular weight oligomers that were not successfully separated in the preceding synthesis step. The peak at 32 minutes could be assigned to the unreacted maleimide 7 due to the excess amount used. Polymer 8 was purified by efficient Soxhlet extraction using methanol, acetone, and THF. The main fraction displayed a number-average molecular weight (*M*_w_) in the range of 125 000–278 000 g mol^−1^, a number-average molecular weight (*M*_n_) of 54 200–92 200 g mol^−1^, and a polydispersity index (PDI) ranging from 2.3 to 3.0, with a high yield of 92%. The efficacy of the purification process for polymer 8 was confirmed through analytical GPC measurement. The GPC spectrum reveals the absence of low molecular weight oligomers and unreacted maleimide residues, indicating successful removal during the purification process (Fig. S2b, ESI†).

The linear-mode MALDI-TOF spectra of the purified polyphenylenes 6 and 8 revealed a periodic peakpattern (Fig. S3 and S4, ESI†). Notably, polyphenylene 6 showed an interval of 683, while polyphenylene 8 exhibited a peak spacing of 1034. These values closely matched the calculated intervals for a repeating unit of each respective polymer. Furthermore, the spectrum of the target polymer 8 displayed smaller-value peaks adjacent to the main peaks, precisely corresponding to an octadodecylmaleimide unit (*m*/*z* ∼ 348), potentially generated *via* a retro-Diels–Alder reaction, initiated by electron collisions during the MALDI-TOF measurement.

In the final step of GNR synthesis, polymer 8 was planarized through oxidative cyclodehydrogenation with a solution of iron(**iii**) chloride (FeCl_3_, 2.76 × 10^−3^ M) at RT for 72 hours, providing AOM-GNR9 in a yield of 92% (Scheme 1). To facilitate additional functionalization, GNR 9 underwent a Sonogashira reaction with ethynyltriisopropylsilane, resulting in the formation of TIPS-GNR10 (also denoted as “clickable” GNR in the following) with a yield of 86%.

**Fig. 1:**
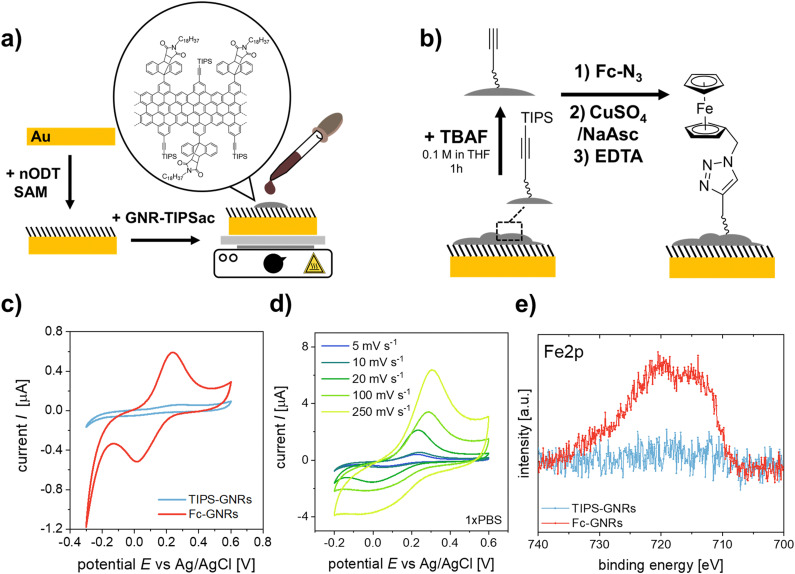
Schematic representation of (a) the fabrication of GNR-TIPS functionalized working electrodes and (b) the click-functionalization thereof with a N_3_-Fc probe. (c) CV curves for GNR-functionalized electrodes before and after click reaction with N_3_-Fc (5 mV s^−1^, PBS 1X). (d) CV curves of Fc-GNR functionalized electrodes at different scan rates (PBS 1X). (e) High-resolution XPS Fe2p signals of GNR-functionalized electrodes before and after clicking of the N_3_-Fc probe.

The as-synthesized “clickable” GNRs were utilized as novel interface for fabricating electrochemical biosensors by depositing them onto Au electrodes (Fig. 1a). To ensure a successful attachment of the GNRs onto the surface, the electrodes were pre-functionalized with a *n*ODT SAM. It has been reported that incubation of a gold surface for 1 hour in a 1 mM *n*ODT solution in EtOH is sufficient to achieve the formation of a SAM with densely packed ODT molecules.^20^ The successful formation of the SAM was confirmed by XPS *via* the monitoring of the high-resolution S2p spectrum (Fig. S11, ESI†). Due to the hydrophobic nature of graphene and graphene-based materials, *e.g.*, GNRs, their adsorption on hydrophobic surfaces, such as *n*ODT-coated electrodes, is favourable.^24^ Despite the edge decoration of the GNRs with AOM groups, achieving solubility remains challenging, potentially limiting its applicability.^25,26^ To perform their deposition on the SAM-functionalized electrodes, the GNRs were solubilized in THF. As previously reported, mild sonication in THF generated a purple dispersions (Fig. S12, ESI†).^26^ GNRs were deposited on the SAM-functionalized electrodes by drop-casting a 1 mg mL^−1^ solution in THF onto the pre-heated substrate. Although the majority of the film appears uniform, there are some noticeable large aggregates present in agreement with other reported GNRs. Nevertheless, even though a lengthy sonication process was applied, aggregates were still present in the suspension and, consequently, on the functionalized electrodes (Fig. S13a, ESI†). It is well known that π-conjugated species, such as GNRs, show self-aggregation through π–π interactions.^27^

Confirmation of the successful deposition of GNRs onto the Au-SAM substrate was achieved through Raman spectroscopy (Fig. S13b, ESI†). In most cases, the Raman spectrum of GNRs closely resembles that of defective graphene.^28,29^ The Raman spectrum of GNRs was deconvoluted using five Lorentzian curves, namely: D, G, D′′, D* and 2D bands (Fig. S13c and d, ESI†).^30,31^ For the preliminary evaluation of the synthesized GNRs, we computed the intensity ratio of the D and G bands (*I*_D_/*I*_G_), and examined the full width at half maximum (FWHM_D_). In accordance with other bottom-up synthesis outcomes, our GNRs display an *I*_D_/*I*_G_ ratio of 1.54 and a FWHM_D_ of 50 cm^−1^, indicating a notable level of disorder compared to GNRs obtained by lithographic approaches.^29^ The *I*_D_/*I*_G_ ratio can be used to unravel the amount of defects present in the GNRs by following the protocol reported by Cançado *et al.*^32^ Initially, the mean defect distance (*L*_D_) was determined by eqn (S1) (see ESI†). GNRs exhibited an *L*_D_ of 9.61 ± 1.10 nm. Since *L*_D_ ≈ 10, we estimated the defect count in each scenario using eqn (S2) (see ESI†), obtaining that the number of defects in the GNRs is between (2.5–4.4) × 10^11^.

To verify the potential for click-functionalization of the deposited GNRs modified with an alkyne group, an azide-bearing redox probe known as ferrocene-azide (N_3_-Fc) was employed. (Fig. 1b).^33–35^ After the successful deprotection of the alkyne-groups, the N_3_-Fc probe was covalently attached *via* the copper(**i**)-catalyzed azide–alkyne cycloaddition (CuAAC) or “click” reaction.^36^ Importantly, the Raman spectrum of GNR remained unaltered upon functionalization with ferrocene (Fig. S13b, ESI†), demonstrating that ferrocene did not increase the number of defects in the GNRs or impact their crystal structure. CV measurements of the ferrocene functionalized GNR electrodes (Fc-GNRs) showed the presence of the redox couple, while the unfunctionalized and TIPS-protected GNR electrode surface (TIPS-GNRs) seemed to be blocked (Fig. 1c). This phenomenon is attributed to bulky TIPS groups that function as a diffusion barrier, impeding the flow of charge between the redox probe and the electrode surface, in agreement with previously reported results on click-functionalized rGO surfaces.^33^ From these results, the amount of functionalized azide moieties can be estimated according to *Γ* = *Q*/*nFA*, where *Q* is the passed charge, *n* is the number of transferred electrons (*n* = 1), *F* is the Faraday constant, and *A* is the electroactive surface area (*A* = 14.5 mm^2^). The averaged charge of the oxidation and reduction of ferrocene attached to the surface correlates to a probe density of 1.1 × 10^−9^ mol cm^−2^. Impressively, this is an order of magnitude higher compared to previously established click-functionalization of graphene-based interfaces and the amount expected from an idealized ferrocene monolayer.^33,37^ Furthermore, signals were recorded at different scan rates (Fig. 1d). Both anodic and cathodic peak currents, *I*_p,a_ and *I*_p,c_, show a linear dependency with the scan rate, *ν*, indicating that electrochemical oxidation/reduction of the monolayer is a surface confined process and, thus, the probe is effectively attached on the electrode surface (Fig. S14, ESI†).^38,39^ The successful click-functionalization was further confirmed by XPS, where only the Fc-GNRs electrodes exhibit a Fe2p component in the recorded spectra (Fig. 1e).

**Fig. 2:**
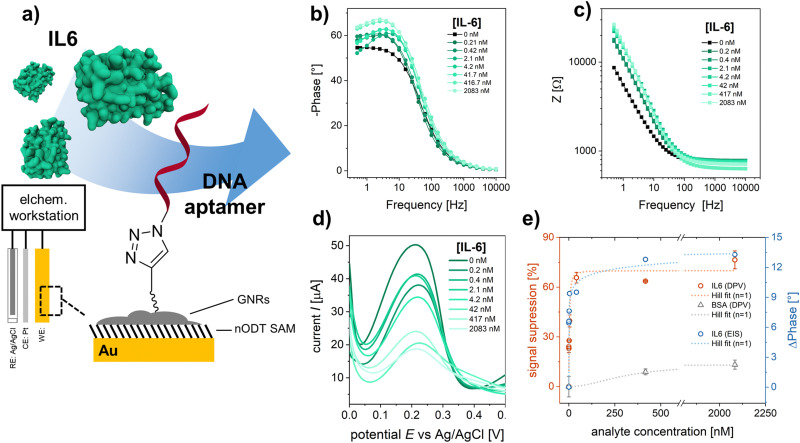
(a) Schematic representation of the developed electrochemical biosensor. EIS spectrum and fitting for the (b) phase angle and (c) impedance after exposing the sensor surface to an increasing IL6 concentration. (d) DPV curves obtained for increasing IL6 concentration. (e) Resulting signals (red: DPV – signal suppression, blue: EIS – phase angle shift at 1 Hz) for specific IL6 binding and unspecific BSA adsorption. Dashed lines indicate the fitting of the signals with the Hill model.

In order to demonstrate the application of the “clickable” GNR interface for electrochemical biosensing, an azide-modified DNA aptamer was clicked onto the GNR-functionalized electrodes (Fig. 2a). The employed aptamer is designed for the detection of IL6, an inflammatory response protein.^40^ EIS was used to evaluate the performance of the developed aptasensor. The electrode surface was exposed to increasing concentrations of IL6, and the electrochemical signals after analyte binding were recorded using [Fe(CN)_6_]^4−^ as the redox probe (Fig. 2b and c). As can be seen in Fig. 2c, the impedance modulus increases with increasing IL6 concentration, especially at low frequencies.^41^ The EIS response was fitted to an equivalent circuit (Fig. S15, ESI†). The evaluated shift in phase angle, ΔPhase, with increasing analyte concentration is shown in Fig. 2e.

To further confirm the analyte binding, DPV was performed for each IL6 concentration (Fig. 2d). A decrease in redox current was observed with increasing IL6 concentration, as the aptamer-IL6 complex formed during recognition acts as a diffusion barrier, hindering the charge transport between the redox probe and the electrode surface.^4,42,43^ The observed decrease in amperometric current was expressed as signal suppression with respect to the measured current in the absence of IL6 (Fig. 2e). Both the results from EIS and DPV are in excellent agreement and were evaluated using Hill's binding model. A zoomed-in version of the response curves for the concentration range below 100 nm is shown in the ESI† (Fig. S16). The obtained signals, *S*, were fitted according to *S* ∝ *θ* = [IL6]^*α*^/(*K*_d_ + [IL6]^*α*^), where *θ* is the faction of recognition sites bound to the target analyte, [IL6] is the IL6 concentration, *K*_d_ is the dissociation constant, and *α* is the Hill coefficient. A *α* = 1 was considered for the signal analysis, corresponding to a non-cooperative binding. For the utilized DNA aptamer, a *K*_d_ of 3.4 ± 0.4 nM and 10.4 ± 3.1 nM were calculated for the binding of IL6 based on the signals obtained from DPV and EIS, respectively, implying a good agreement between the two different approaches. For the IL6 response curves the sensor performance was evaluated and the obtained performance factors are summarized in Table S2 (ESI†). The selectivity of the sensor to IL6 was evaluated by using BSA, which has a strong tendency to adsorb to surfaces non-specifically and is generally used as a negative control in biosensing studies.^44^ At saturation concentrations (2 μM), a signal suppression of only ∼15% was observed for BSA as analyte, confirming the selective detection of IL6 by the employed aptamer.

The developed electrochemical aptasensor demonstrates the great potential of the “clickable” GNRs as biointerface for specific analyte detection. Nonetheless, a label-free detection mechanism would offer significant advantages for the application in healthcare, particularly in PoC devices. In this context, electrolyte-gated field-effect transistors (EG-FETs) have emerged as a highly promising technology.^45^ Among various materials, EG-FETs based on rGO have demonstrated great potential for their application in biosensing.^31,46^

To preserve its chemical structure and electrical properties, rGO functionalization is commonly performed through non-covalent means, such as *via* π–π interactions with 1-pyrenebutanoic acid succinimidylester (PBASE).^47,48^ The extended π-system of the GNR compared to conventional pyrene linkers leads to stronger interactions and, therefore, increased stability of the attached interface. This comes with the trade-off of a more costly and complex synthesis compared to existing, pyrene-based linkers. On the other hand, the combination with alkyne-modifications of the GNRs enables a covalent and orthogonal immobilization of biomolecules *via* click chemistry with a high probe density.

**Fig. 3:**
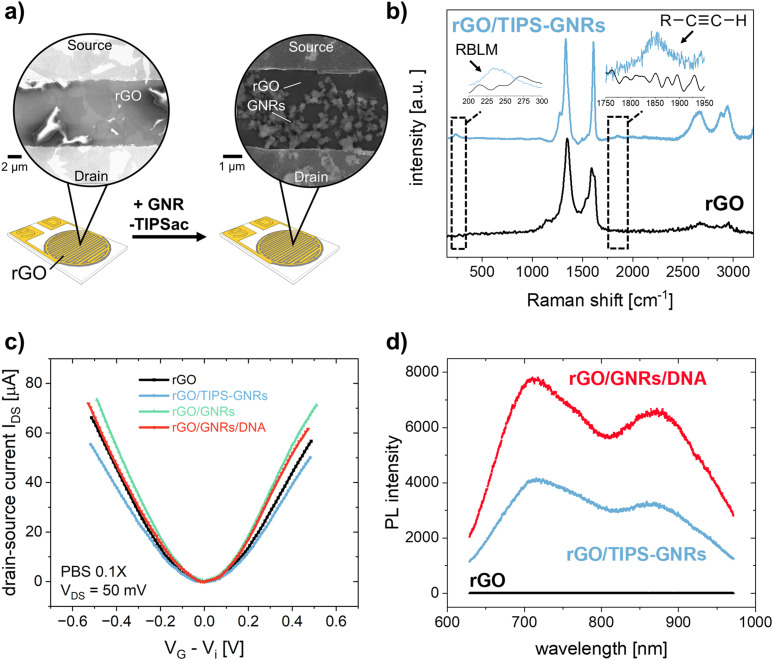
(a) Schematic representation of the rGO-based FET before and after functionalization with GNR-TIPS. Zoom-in shows SEM images of the transistor channel. (b) Raman spectrum of pristine rGO (black) and after functionalization with GNR-TIPS (blue). Insets show zoomed-in parts of the spectrum representing the RBLM and alkyne band. (c) Dirac-point corrected *I*_DS_*V*_G_ transfer characteristics of the FET (*V*_DS_ = 50 mV, PBS 0.1X) and (d) PL spectrum of the transistor channel recorded at different functionalization steps.

rGO-based FETs were fabricated using substrates bearing 90 pairs of interdigitated gold electrode (IDE) arrays, each with a width of 5 μm and a channel length of 5 μm.^23^ GNRs were deposited onto the rGO surface by drop casting the GNR dispersion in THF onto pre-heated FET substrates (see Experimental section). Fig. 3a shows the SEM images of the rGO transistor channel before and after GNR deposition. Fig. 3b shows the Raman spectrum of rGO in full agreement with other reported rGO.^31^ After the non-covalent functionalization of rGO with TIPS-GNRs, the Raman spectrum is dominated by the signals of GNRs (Fig. 3b, blue spectrum). A distinct indication for the successful deposition of GNRs on the rGO surface is the presence of a ribbon width-specific low-frequency mode at ∼235 cm^−1^ (Fig. 3b, inset). This so-called radial breathing-like mode (RBLM) is characteristic of the GNR structure and can be estimated roughly by the width of the GNR (see ESI† for details).^26,49^ With a maximum width of 1.13 nm for the synthesized GNRs,^26^ without considering the solubilizing side chains, the RBLM amounted to 285 cm^−1^, which is in good agreement with the Raman experimental results. The shift to higher wavenumbers can be attributed to a deformation in the GNR edges, caused by the presence of AOM groups. Notably, within the 1800–1900 cm^−1^ range, the signal corresponding to the triple bond of the attached alkyne-group is distinctly discernible (Fig. 3b, inset).^50,51^

To verify the electronic performance before and after GNR functionalization, *I*_DS_*V*_DS_ output and *I*_DS_*V*_G_ transfer characteristics were recorded. Generally, the curvature of the output curves indicates the quality of the contacts between the active transistor material and the electrodes as well as the charge transport within the channel.^5^ The output curve for GNR-TIPS functionalized rGO FET devices showed a linear Ohmic regime at an applied *V*_DS_ of 0 to 500 mV for an investigated *V*_G_ range between −500 and 500 mV (Fig. S17a, ESI†), which indicates an excellent electrical contact (a *V*_DS_ of 50 mV was applied for all experiments according to the linear regime). Fig. 3c shows the Dirac point-corrected *I*_DS_*V*_G_ transfer curves obtained from FET devices with pristine rGO, after GNR-TIPS deposition, after deprotection of the alkynyl moieties on the periphery of the immobilized GNRs, and after click-functionalization thereof with the DNA aptamer. Typical ambipolar transfer curves, with a hole (h^+^) and electron (e^−^) accumulation branch, as expected for rGO-based devices, were obtained. The minimum, denoted as Dirac point (*V*_i_), is associated with the gate voltage at the minimum of the transfer curve.

The deposition of GNRs on the rGO surface leads to a reduction in transconductance (*g*_m_) (the slope of the linear part of the h^+^ and e^−^ accumulation branch). We hypothesize that the immobilized GNRs introduce additional scattering sites due to the high number of defects, affecting the charge carrier mobility and thus reducing *g*_m_ (Fig. S17b, ESI†).^5^ Additionally, the functionalization of the rGO surface with GNRs containing bulky TIPS protection groups results *via* capacitive coupling in shallower slopes for both the h^+^ and e^−^ accumulation branches of the transfer curve (Fig. 3c). After TIPS-deprotection by exposing the FET device to a TBAF solution in THF, the slopes (h^+^ and e^−^) of the transfer curve are restored, attributed to the removal of the bulky TIPS protection group. This is in agreement with the observations made for CV measurements, as discussed above, and previously reported results.^43^ Further click-functionalization with the DNA aptamer leads to only a slight reduction in *g*_m_*via* capacitive coupling due to the formation of the biointerface. It is expected that the DNA aptamers are more sparsely attached compared to the bulky TIPS groups due to steric and electrostatic repulsion, resulting in a less prominent influence on the slopes of the transfer curve.

The most common electrical metric to observe in gFETs is the change of the Dirac point (*V*_i_) which depends on the doping level of the graphene.^5,52^ Changes at the surface of graphene can alter its doping state, thus creating a negative or positive shift of the Dirac point, depending on the positive or negative charge of the immobilized species.^52^ Fig. S17c (ESI†) shows the change of Dirac point upon the different functionalization steps. The deposition of GNR-TIPS on the rGO surface led to a slight positive shift of the Dirac point. It has been shown that aromatic molecules can n-dope 2D materials, such as MoS_2_, due to charge transfer and/or dipolar interactions.^53^ Several studies have reported a positive Dirac point shift for the immobilization of PBASE on graphene-based FET channels *via* π–π interactions.^54–57^ The deprotection of TIPS, on the other hand, led to a p-doping of the device.^43^ More importantly, the immobilization of the highly negatively charged DNA aptamer led to a significant positive Dirac point shift associated with a n-type doping of the device through the field-effect.^52^

Interestingly, upon deposition of the GNRs onto the rGO surface, a PL band appeared (Fig. 3d). Compared to graphene, the structural confinement into nanometer-wide strips, *i.e.* GNRs, opens up a band gap in the graphene electronic structure that can be tuned by their width and edge configuration.^58,59^ Although the resulting PL signal is quenched if the GNRs are applied on metallic substrates, such as the electrochemical working electrodes, PL can be detected at the interface, allowing for a dual-readout.^60^ Consequently, the distribution of GNRs can be obtained *via* PL mapping on the transistor channel (Fig. S18, ESI†). More importantly, the resulting PL signal is sensitive to the GNR functionalization. While the subsequent attachment of the DNA aptamer at the edge of the GNRs did not result in any specific alterations in their Raman spectrum, an abrupt increase in PL was observed (Fig. 3d). PL offers a great alternative to other forms of optical sensing technologies, providing amongst other advantages the possibility for high sensitivity, multiparametric measurements, and imaging. Various materials have been developed, among which fluorescent dyes are usually the material of choice for many applications.^61^ However, this typically requires an attachment to the target molecule which may alter the interaction with the receptor. In our case the interface itself presents the PL property, rendering a labelling of the analyte unnecessary, allowing a broader range of applications. Recently, the potential of utilizing the PL property, integrated in the sensor interface, has been demonstrated by using graphene quantum dots embedded in a nanofluidic membrane for a rapid and reliable optical readout of pH changes, associated with human health monitoring.^62^ In our work, the PL signal of the GNRs opens up the possibility for direct and simultaneous optical and electronic probing of the biointerface immobilized on the transistor channel, offering a compelling alternative to previously established, multivariable sensor configurations based on FET and surface plasmon resonance.^63,64^ As a next step, an integration of both sensing principles into one FET measurement cell is foreseen, to better investigate and understand the obtained signals, and to demonstrate the simultaneous, optical-electronic analyte detection. We believe that the combination of electronic and optical readouts can offer particular significance when studying interfacial processes (*e.g.*, occurring in biosensing), as shown in previously established dual-mode sensing platforms.^63^ Therefore, the use of “clickable” GNRs as the linker layer holds great promise for application as a multivariable interface in next-generation FET-based biosensing devices.

## Conclusions

In conclusion, our study demonstrates the versatile and promising utility of “clickable” GNRs, obtained *via* an efficient solution synthesis, as the interface in the field of electrochemical biosensors. The immobilized GNRs allow for a direct and covalent attachment of the biorecognition unit, *e.g.* a DNA aptamer, with a high probe density. Additionally, in the domain of optoelectronic FETs, the use of GNRs as an interface was equally compelling. The deposition of GNRs onto rGO FETs offers a more robust and stable attachment of the biointerface due to the extended π-system compared to conventional aromatic linker molecules without compromising the quality of the transistor channel. Notably, the GNR interface introduced a PL signal, offering an innovative avenue for direct and simultaneous optical and electronic probing of the biointerface applied in graphene-based FETs. The application of GNRs plays a crucial role in advancing biosensing technologies and device innovation, with the potential to improve point-of-care diagnostics and advanced sensor systems for healthcare applications.

## Author contributions

The manuscript was written through the contributions of all authors. All authors have given approval to the final version of the manuscript. Conceptualization: R. H., G. E. F., A. G., W. K., K. M.; validation: R. H., G. E. F., A. G., V. M.-G., C. V., Z. Q.; formal analysis: R. H., G. E. F., A. G., V. M.-G., C. V., Z. Q., W. K., K. M.; investigation: R. H., G. E. F., A. G., V. M.-G., C. V., Z. Q.; writing – original draft: R. H., G. E. F., A. G, V. M.-G., C. V.; writing – review & editing: Z. Q., W. K., K. M., P. S., C. K.; visualization: R. H., G. E. F., A. G., V. M.-G.; supervision: W. K., K. M., P. S.; project administration: W. K., C. K.; funding acquisition: W. K., C. K.

## Conflicts of interest

There are no conflicts to declare.

## Supplementary Material

NH-009-D3NH00590A-s001
