# Supplementary material for: “Clickable” graphene nanoribbons for biosensor interfaces
Source: Nanoscale Horiz. 2024 Feb 22;9(4):598–608. doi: 10.1039/d3nh00590a (PMC10962640; doi:10.1039/d3nh00590a)
Supplement: NH-009-D3NH00590A-s001 [file NH-009-D3NH00590A-s001.pdf]

Electronic Supplementary Material (ESI) for Nanoscale Horizons.
This journal is © The Royal Society of Chemistry 2024

SUPPORTING INFORMATION

"Clickable" Graphene Nanoribbons for Biosensor Interfaces

Roger Hasler[†a,b]*, Gonzalo Fenoy[†a,c], Alicia Götz[d], Verónica Montes-García[e], Cataldo Valentini[e,f], Zijie Qiu[d,g], Christoph Kleber[b], Paolo Samorì[e], Klaus Müllen[d], Wolfgang Knoll[a,b]*

[a] AIT Austrian Institute of Technology GmbH, 3430 Tulln, Austria
[b] Laboratory for Life Sciences and Technology (LiST), Faculty of Medicine and Dentistry, Danube Private University, 3500 Krems, Austria, Email: wolfgang.knoll@dp-uni.ac.at; roger.hasler@dp-uni.ac.at
[c] Instituto de Investigaciones Fisicoquímicas Teóricas y Aplicadas (INIFTA), Departamento de Química, Facultad de Ciencias Exactas, Universidad Nacional de La Plata, La Plata B1904DPI, Argentina
[d] Max Planck Institute for Polymer Research, Ackermannweg 10, D-55128 Mainz, Germany
[e] Université de Strasbourg, CNRS, Institut de Science et d'Ingénierie Supramoléculaires, 8 allée Gaspard Monge, 67000 Strasbourg, France
[f] Centre for Advanced Technologies, Adam Mickiewicz University, Uniwersytetu Poznańskiego 10, 61-614 Poznań, Poland
[g] School of Science and Engineering, Shenzhen Institute of Aggregate Science and Technology, The Chinese University of Hong Kong, Shenzhen (CUHK-Shenzhen), Guangdong 518172, P.R. China

Synthetic information

1-(4-(Anthracen-9-yl)phenyl)-3-(4-iodophenyl)propan-2-one (2)

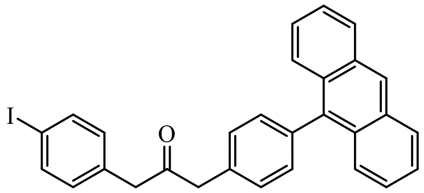

1,3-Bis(4-iodophenyl)propan-2-one (**1**) (2.5 g, 5.4 mmol, 1 eq), anthracene-9-yl-boronic acid (1.2 g, 5.4 mmol, 1 eq) and tetrakis(triphenylphosphine)palladium(0) (125 mg, 0,108 mmol, 0.02 eq) were dissolved in THF (50 mL) and an aqueous solution of potassium carbonate (10 mL, 3.0 g, 21.6 mmol, 4 eq) was added. The mixture was stirred overnight at 60 °C.

The reaction mixture was subsequently cooled to room temperature, and the organic components were extracted using dichloromethane. The combined organic phases underwent drying with sodium sulphate, followed by solvent removal through distillation using a rotary evaporator. The resulting crude product was further purified through column chromatography, employing silica gel as the stationary phase (eluent: 33% dichloromethane in hexane). The desired product was isolated as a yellowish solid (1.3 g, 47%). ^{1}H NMR (300 MHz, CD_2Cl_2) δ = 8.52 (s, 1H); 8.07 (d, J = 8.4 Hz, 2H); 7.73 - 7.62 (m, 4H); 7.51 - 7.44 (m, 2H); 7.41 - 7.33 (m, 6H); 7.00 (d, J = 8.2 Hz, 1H); 3.93 (s, 2H); 3.84 (s, 2H). ^{13}C NMR (75 MHz, CD_2Cl_2) δ = 205.10, 138.07, 137.84, 136.93, 134.44, 133.72, 132.11, 132.01, 131.80, 130.57, 130.04, 128.71, 127.01, 126.96, 125.83, 125.54, 92.68, 49.66, 48.88. HRMS (MALDI-TOF, positive) *m/z*: Calcd for $C_{29}H_{21}IO$: 512.0637; found 512.0644 $[M]^+$.

2-(4-(Anthracen-9-yl)phenyl)-5-(4-iodophenyl)-3-phenyl-4-(3-((triisopropylsilyl)ethynyl)phenyl)cyclopenta-2,4-dien-1-one (**4**)

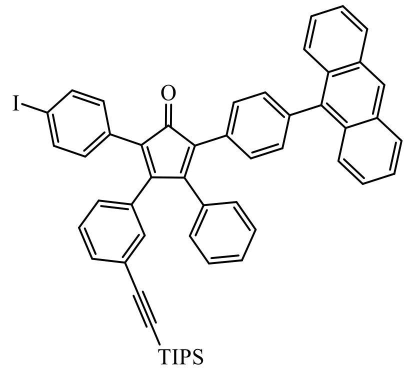

Compound **S2** (330 mg, 0.65 mmol) and 3-((triisopropylsilyl)ethynyl)benzil (258 mg, 0.661 mmol) were dissolved in *tert*-butanol (30 mL) and heated to 65°C. Tetrabutylammonium hydroxide (40% in methanol, 170 mg, 0.19 mL, 0.26 mmol) was then added and the reaction was stirred at 95°C for 1 hour. The reaction mixture was cooled to room temperature, quenched by an aqueous HCl solution (2 M, 0.13 mL) and extracted with dichloromethane. The combined organic phases were washed twice with water and dried over sodium sulphate. The product was purified by silica column chromatography (eluent: 10% dichloromethane in hexane), and obtained in the form of a viscous, violet oil (445 g, 79%): ^{1}H NMR (700 MHz, CD_2Cl_2) δ = 8.51 (s, 1H), 8.06 (d, J = 8.4 Hz, 2H), 7.73 - 7.59 (m, 4H), 7.53 - 7.44 (m, 4H), 7.42 - 7.16 (m, 9H), 7.14 - 6.95 (m, 6H), 1.13 - 1.05 (m, 21H). 13C NMR (176 MHz, CD_2Cl_2) δ = 200.28, 200.25, 156.00, 155.34, 154.68, 154.21, 138.63, 138.47, 137.74, 137.65, 136.97, 136.93, 133.63, 133.45, 133.37, 133.34, 133.31, 133.19, 132.29, 132.20, 131.79, 131.51, 131.39, 130.85, 130.68, 130,48, 130,43, 130,22, 129,79, 129,71, 129,62, 129,51, 129,34, 129,29, 128,70, 128,66, 128,61, 127,05, 126,07, 125,88, 125,84, 125,76, 125,55, 125,30, 124,82, 123,82, 106,70, 106,64, 94,14, 93,94, 92,03, 91,81, 18,82, 11,69. HRMS (MALDI-TOF, positive) *m/z*: Calcd for $C_{54}H_{47}IOSi$: 866,2441; Found: 866,2463 $[M]^+$.

2-(4-(Anthracen-9-yl)phenyl)-4-(3-ethynylphenyl)-5-(4-iodophenyl)-3-phenylcyclopenta-2,4-dien-1-one (**5**)

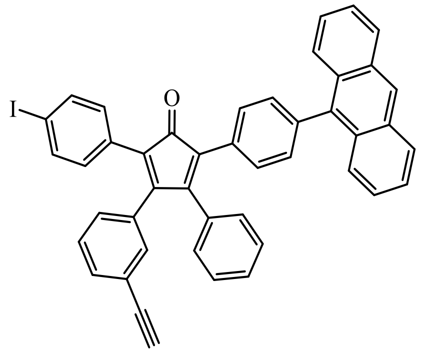

To a solution of 2-(4-(Anthracen-9-yl)phenyl)-5-(4-iodophenyl)-3-phenyl-4-(3-((triisopropylsilyl)ethynyl)phenyl)cyclopenta-2,4-dien-1-one (**4**) (345 mg, 0.401 mmol, 1.0 eq) in dichloromethane (15 mL) tetra-*n*-butylammonium fluoride (1M, 110 mg 0.42 mL, 0.42 mmol, 1.01 eq)

was added dropwise. After stirring at room temperature for 20 minutes, water was added to the reaction mixture and then the organic compound was extracted three times with dichloromethane. Subsequently, the combined organic phases were dried over magnesium sulphate. The solvent was removed *in vacuo*, and the residue was purified by column chromatography over silica gel (eluent: 25% dichloromethane in hexane). The product was obtained as a purple, viscous solid (190 mg, 67%): ^{1}H NMR (300 MHz, CD_2Cl_2) δ = 8.51 (s, 1H), 8.06 (d, *J* = 8.4 Hz, 2H), 7.69 - 7.62(m, 4H), 7.49 - 7.22 (m, 13H), 7.13 - 6.99 (m, 6H), 3.11 (s, 1H). ^{13}C NMR (75 MHz, CD_2Cl_2) δ = 200.16, 200.08, 155.52, 155.57, 154.58, 154.14, 138.63, 138.43, 137.70, 137.26, 136.90, 136.85, 133.77, 133.68, 133.34, 133.09, 133.0, 132.75, 132.70, 132.60, 132.25, 132.22, 131.72, 131.45, 131.37, 131.37, 130.74, 130.42, 130.39, 130.32, 130.25, 130.13, 130.0, 129.70, 129.50, 129.31, 129.23, 128.78, 128.64, 128.60, 128.57, 127.01, 126.99, 126.97, 126.96, 126.53, 125.84, 125.81, 125.65, 125.67, 125.53, 125.32, 124.85, 122.53, 122.45, 94.18, 93.97, 83.11, 83.01, 78.19, 77.94. HRMS (MALDI-TOF, positive) *m/z*: Calcd for $C_{45}H_{27}IO$: 710.1113; Found: 710.1107 $[M]^+$.

Synthetic protocol of the polyphenylenes and GRN

Anthracene-Polyphenylene (**6**)

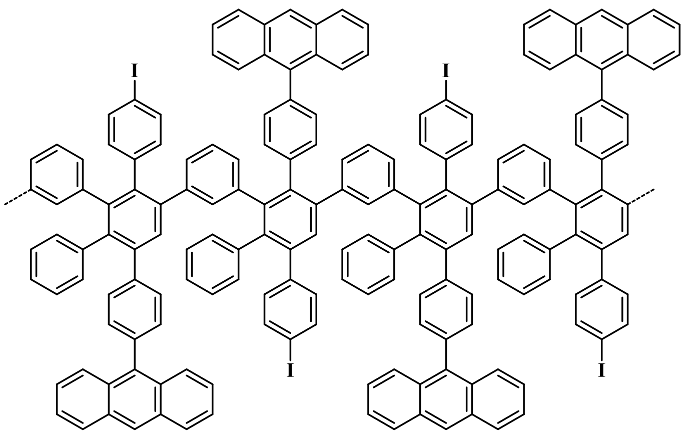

In a Schlenk tube, 2-(4-(anthracen-9-yl)phenyl)-4-(3-ethynylphenyl)-5-(4-iodophenyl)-3-phenylcyclopenta-2,4-dien-1-one (**5**) (334 mg, 0.47 mmol, 10-6 mM) was dissolved in diphenyl ether (1 mL) and the solution was degassed with argon. The reaction mixture was heated to 250°C and stirred for 36 hours. For purification, the yellowish residue was dissolved in a little THF and precipitated in methanol. The residue was washed with sufficient methanol, then redissolved in THF and again precipitated in methanol. The precipitate was filtered off, washed with methanol and purified by Soxhlet extraction (methanol, acetone, ethyl acetate, THF). However, the low-molecular oligomers could not be completely separated, so the mixture was used in the next step without further purification (255 mg, 79%).

AOM-PP (**8**)

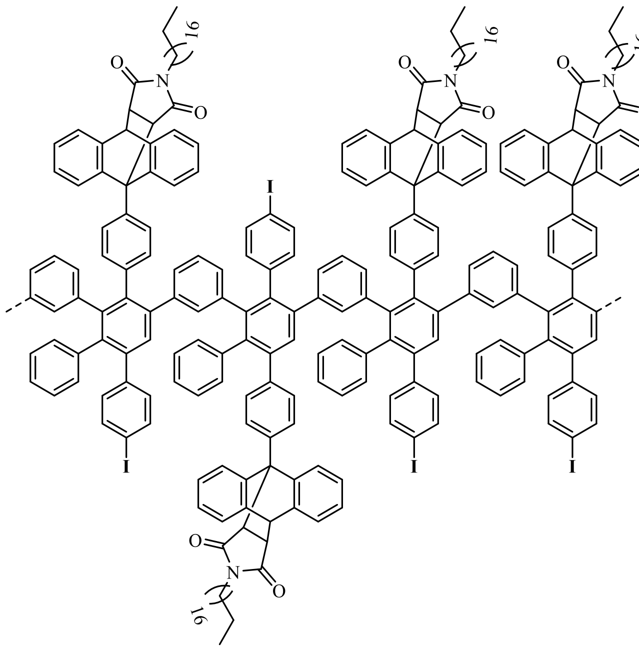

Polyphenylene **6** (100 mg, 0.147 mmol, 1 eq) and *N*-*n*-octadecylmaleimide (512 mg, 1.46 mmol, 10 eq) were dissolved in anhydrous and oxygen-free o-xylene (9 mL) and stirred at 150°C for 36 hours. For purification, the reaction mixture was cooled and added to methanol (100 mL). The precipitate was filtered, washed with methanol and purified by Soxhlet extraction (methanol, acetone, THF). The polymer was isolated in the form of a shiny yellow solid (103 mg, 92 %). GPC (THF) THF fraction: M_w = 125 000–278 000 g mol^{-1}; M_n = 54 200 - 92 200 g·mol^{-1}, PDI = 2.3–3.0.

AOM-GNR (**9**)

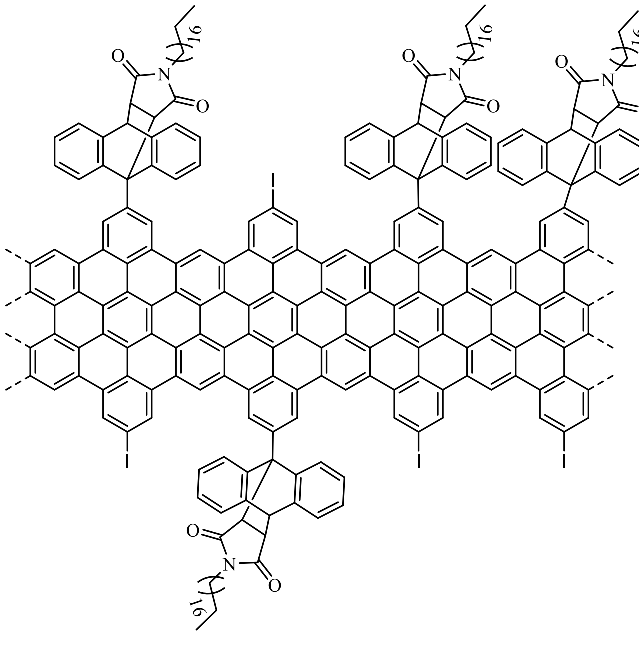

Polymer **8** (66 mg, 0.066 mmol, 1 eq) was dissolved in unstabilized dichloromethane (280 mL). The suspension of iron(III) chloride (894 mg, 5.51 mmol, 84 eq) in nitromethane (2 mL) was added and the

mixture was stirred at room temperature for 72 hours. The reaction mixture was terminated with the addition of methanol and the precipitates were filtered with a membrane filter (PTFE, 200 nm). The precipitate was washed with sufficient methanol and THF. For purification, the solid was treated again in THF in an ultrasonic bath, precipitated in methanol and filtered off. This procedure was repeated several times and the product was obtained as a dark purple solid (60 mg, 92%): UV-vis (THF), λ_{max}/nm: 542. FTIR (powder, cm^{-1}): 2921, 2914, 2846, 2221, 2210, 2204, 2199, 2191, 2187, 2176, 2171, 2160, 2153, 2146, 2136, 2127, 2110, 2095, 2075, 2066, 2058, 2044, 2032, 2026, 2016, 2005, 1997, 1998, 1981, 1978, 1972, 1962, 1695, 1652, 1559, 1458, 1396, 1343, 1134, 852, 817, 763, 749, 717, 644, 620, 558. Raman (powder, cm^{-1}): 1327, 1604, 2672, 2927, 3210.

TIPS-GNR (**10**)

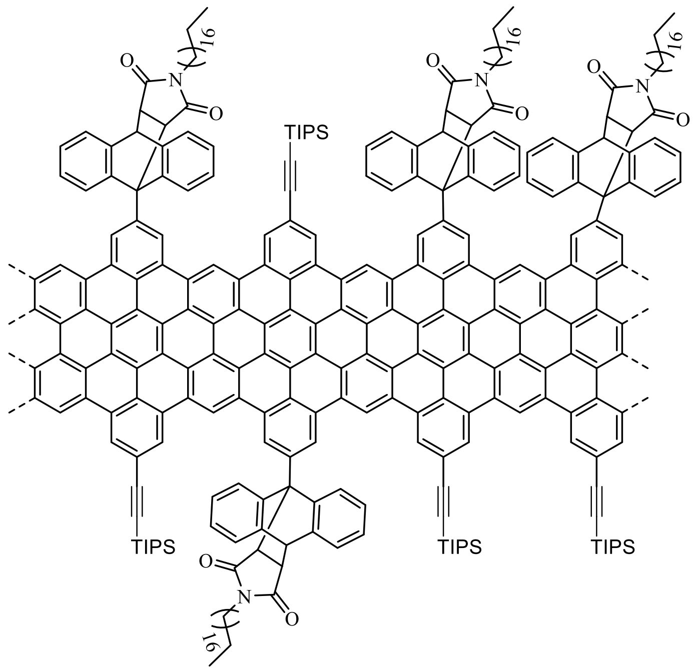

GNR **9** (23.0 mg, 22.6 µmol) and CuI (0.429 mg, 2.25 µmol, 0.1 eq) were dissolved in a solvent mixture of dry and oxygen-free THF (9 mL)and NEt_3 (6 mL). The dispersion was treated by sonication for 5 minutes and then the catalyst $Pd(PPh_3)_4$ (3.91 mg, 3.38 µmol, 0.15 eq) was added under argon flow. The mixture was degassed for 10 minutes, followed by the addition of ethynyltriisopropylsilane (20.6 mg, 0.113 mmol, 5 eq). The reaction mixture was stirred at 70 °C for 72 hours. The solution was cooled to room temperature, and the resulting material was subjected to filtration and subsequently washed with tetrahydrofuran (THF) and methanol. The GNR was then dissolved in THF and underwent a 10-minute sonication, followed by filtration and additional washes. This entire process was repeated multiple times to thoroughly eliminate any remaining impurities. The desired GNR was yielded as a dark violet powder (21 mg, 86%). The GNR was used without further characterization in the next synthetic step.

Analytical information

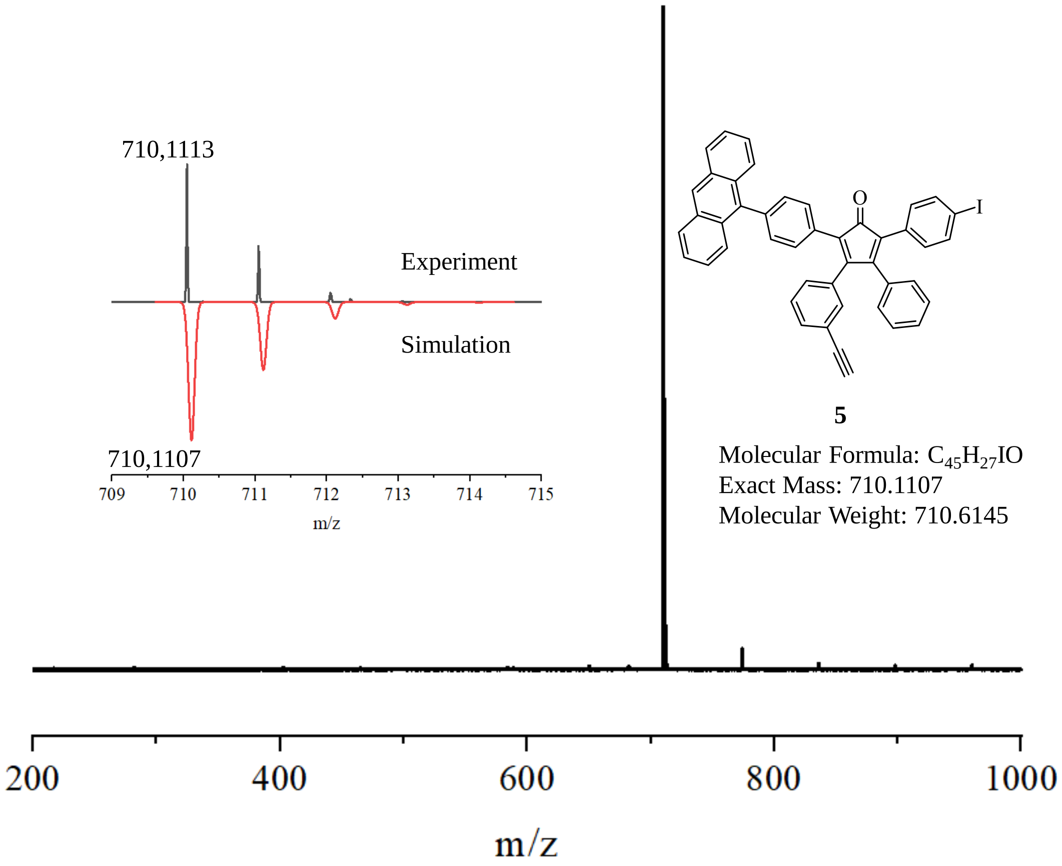

Figure S1. High-resolution MALDI-TOF mass spectrum and isotopic distribution pattern of compound **5** (DCTB).

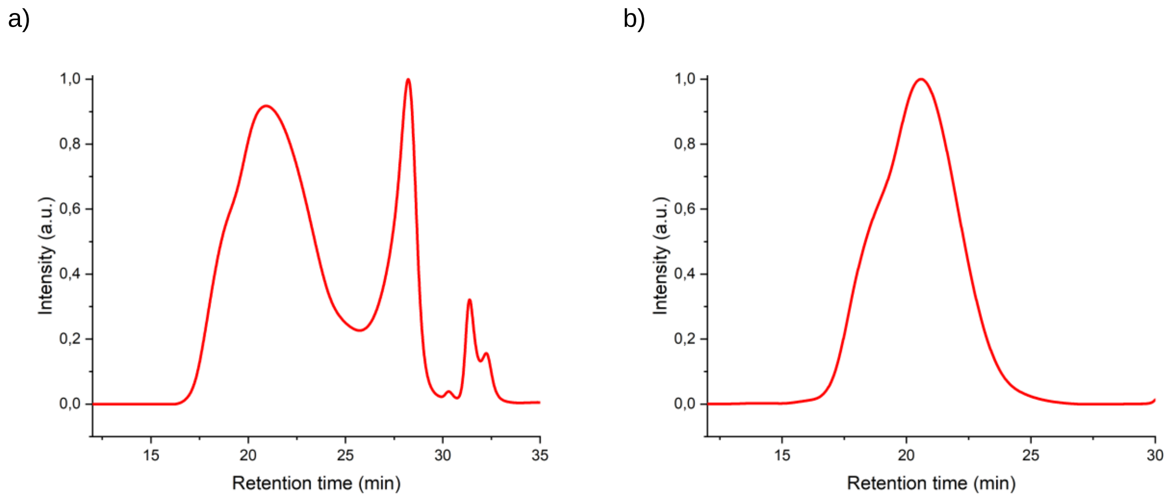

Figure S2. Normalized SEC profiles of polyphenylene precursor **8** (eluent: THF, 1.0 mL min^{-1}, UV detector) before (a) and after (b) the fractionation by the preparative SEC.

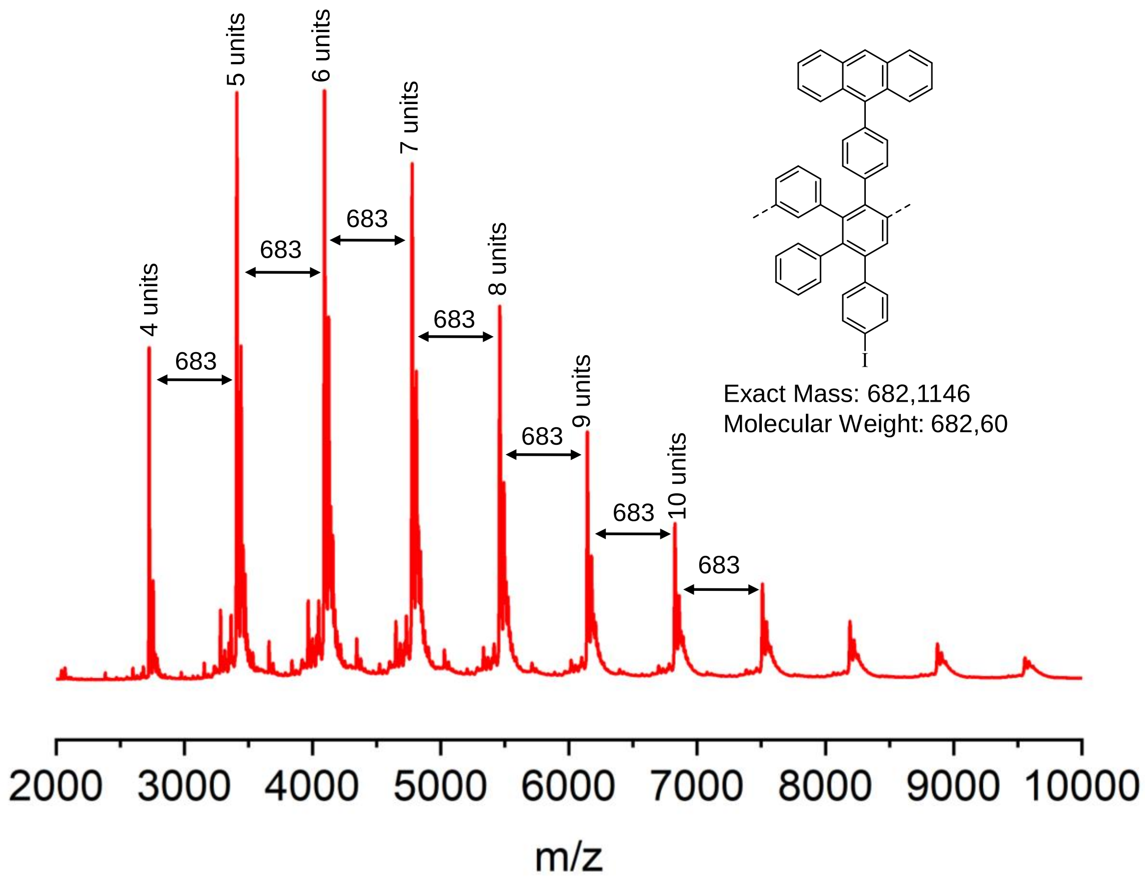

Figure S3. Linear-mode MALDI-TOF analysis of compound **6** (matrix: DCTB).

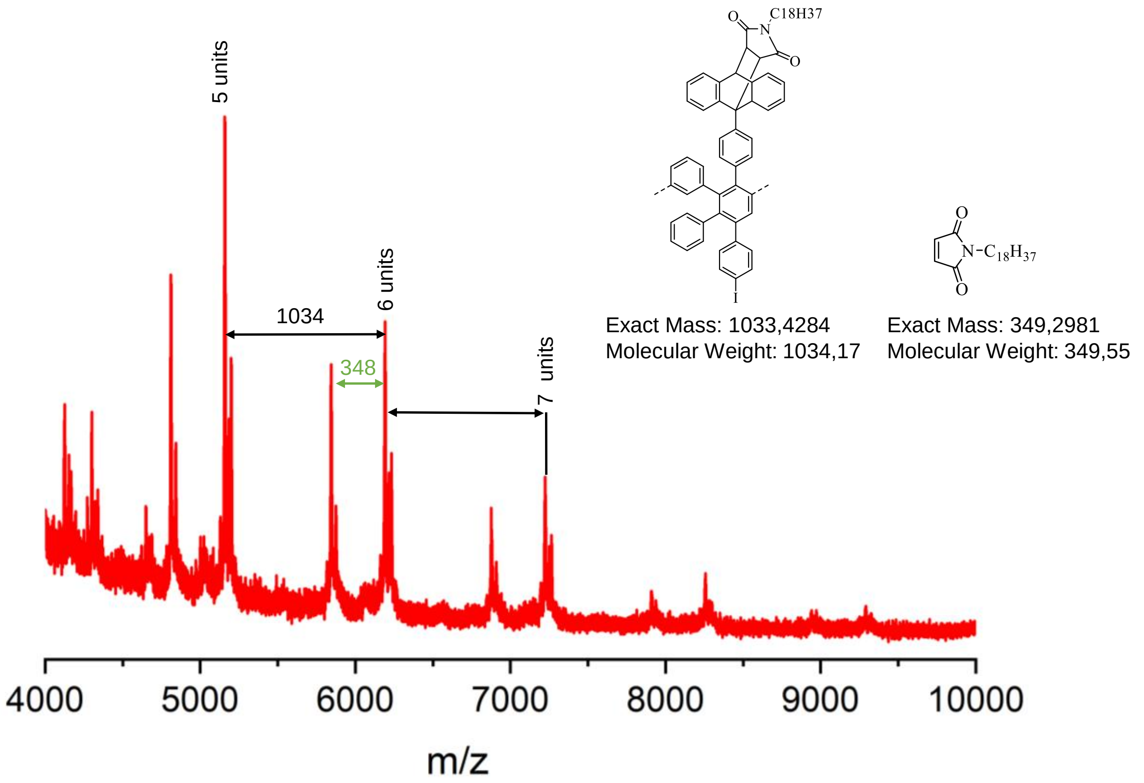

Figure S4. Linear-mode MALDI-TOF analysis of compound **8** (matrix: DCTB).

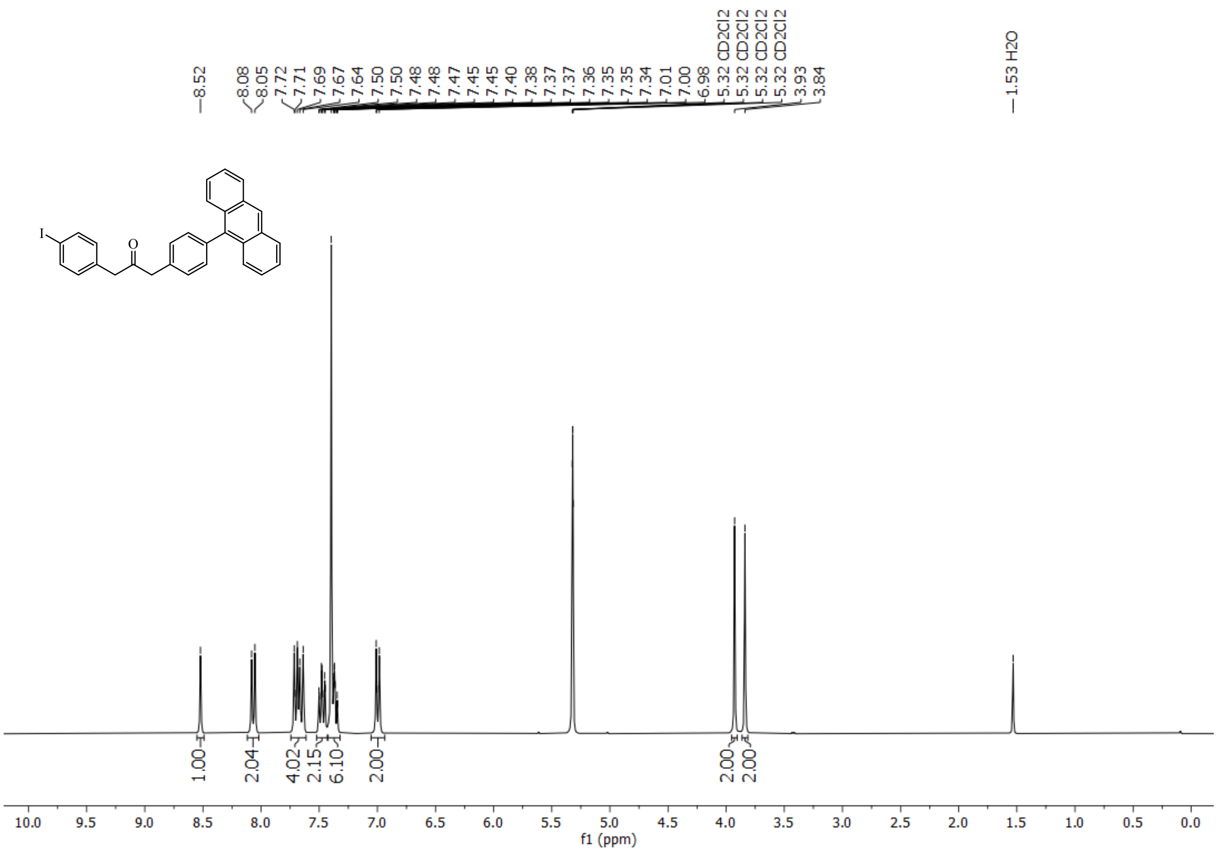

Figure S5. ^{1}H NMR spectrum of compound **2** (300 MHz, CD_2Cl_2, 298K).

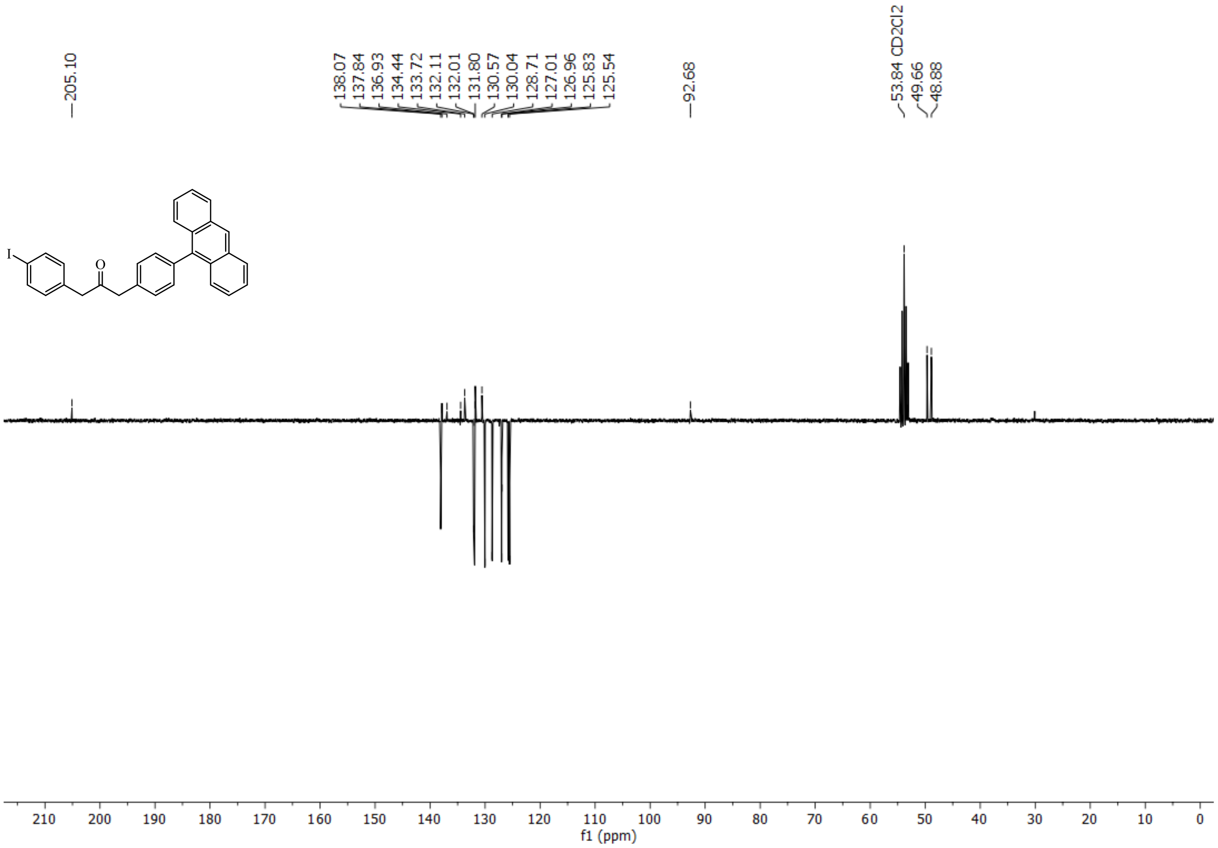

Figure S6. ^{13}C NMR Spectrum of compound **2** (75 MHz, CD_2Cl_2, 298K).

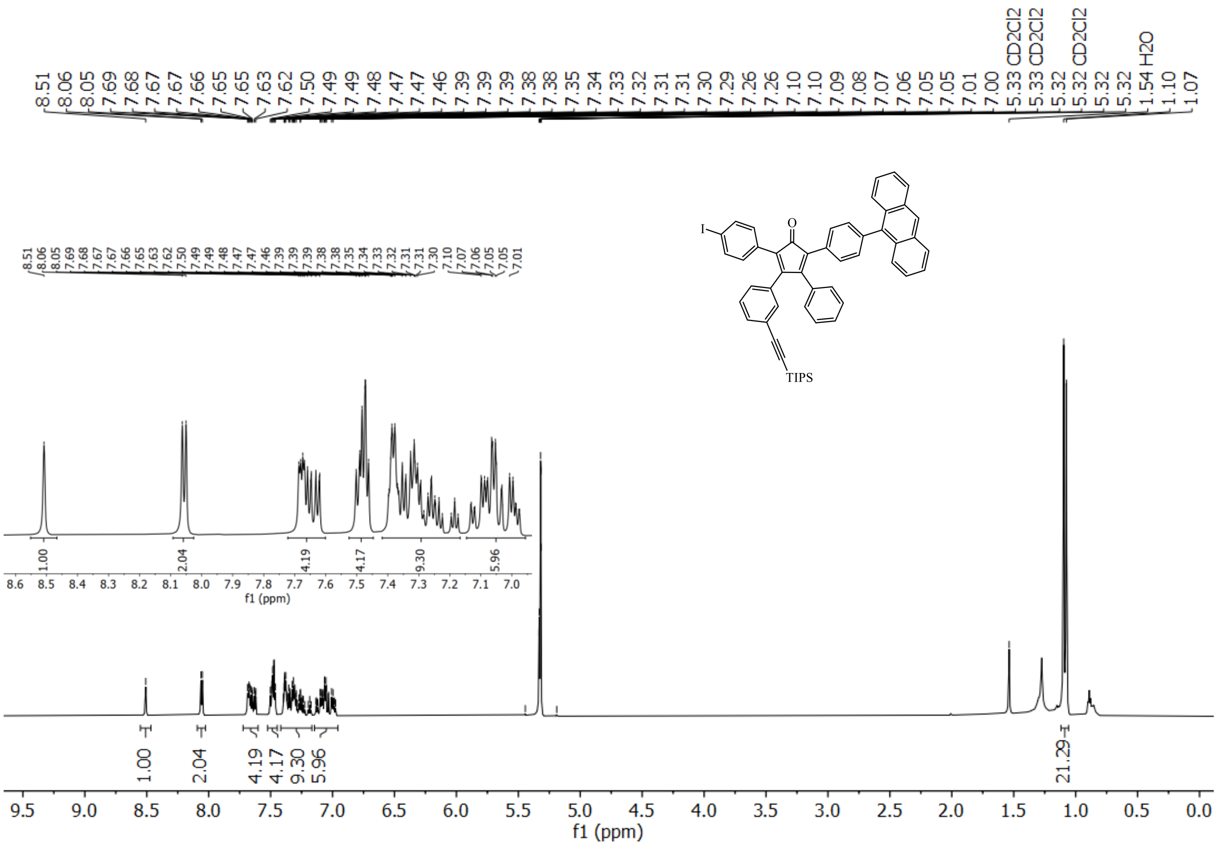

Figure S7. ^{1}H NMR spectrum of compound **4** (700 MHz, CD_2Cl_2, 298K).

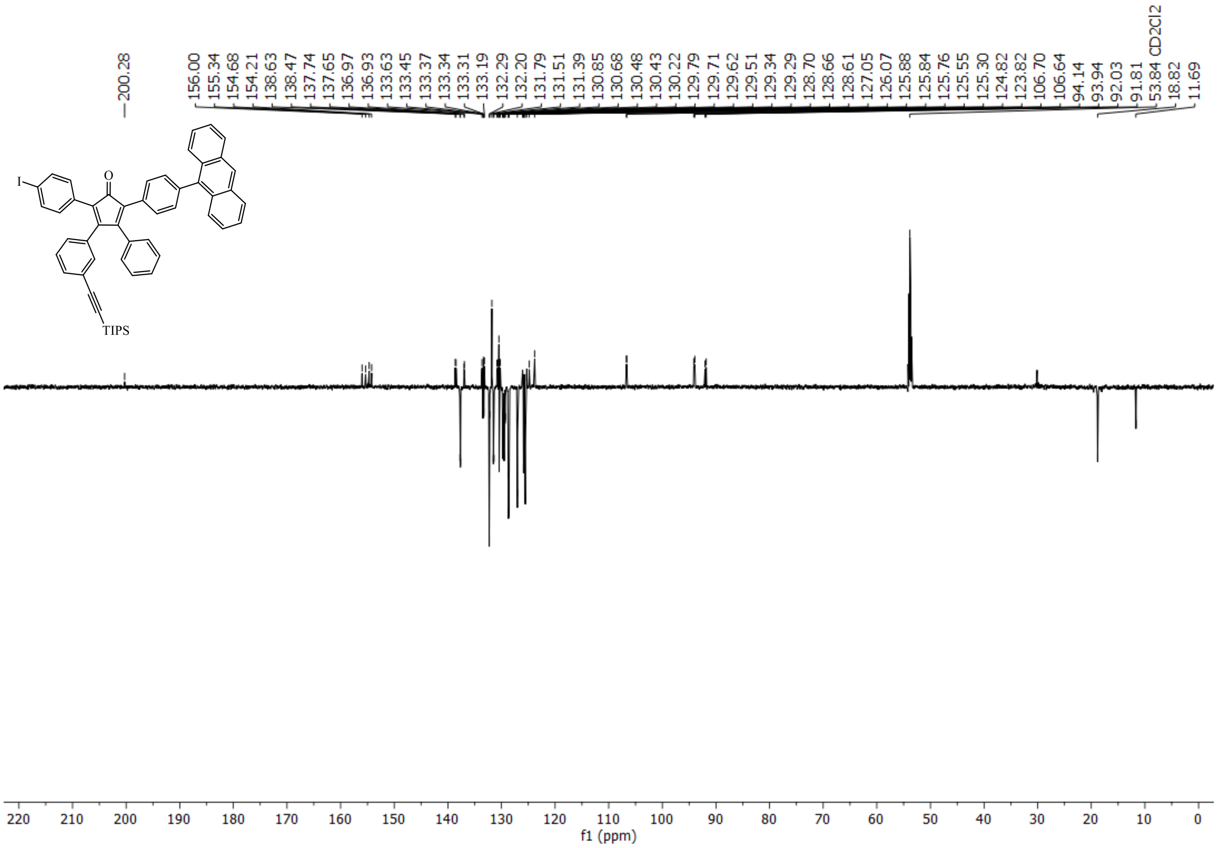

Figure S8. ^{13}C NMR spectrum of compound **4** (176 MHz, CD_2Cl_2).

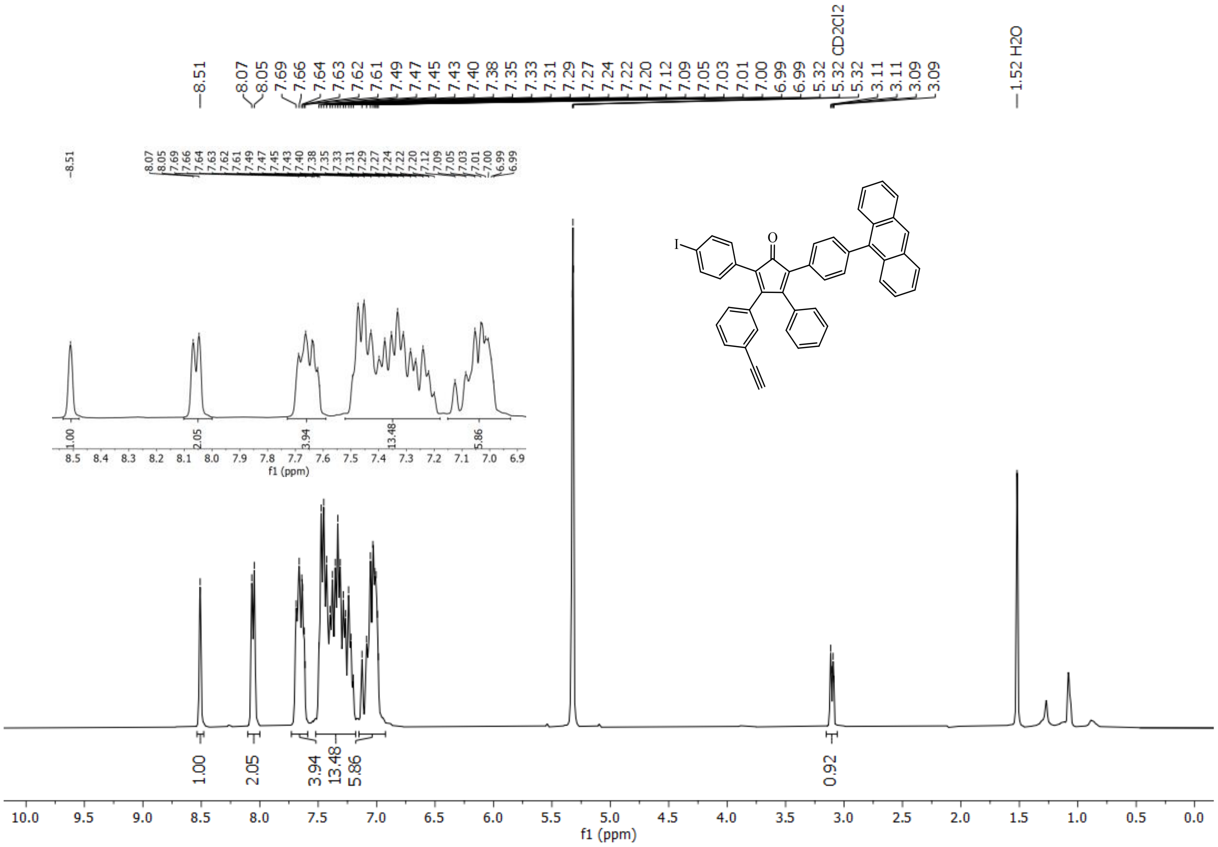

Figure S9. ^{1}H NMR spectrum of compound **5** (400 MHz, CD_2Cl_2, 298K).

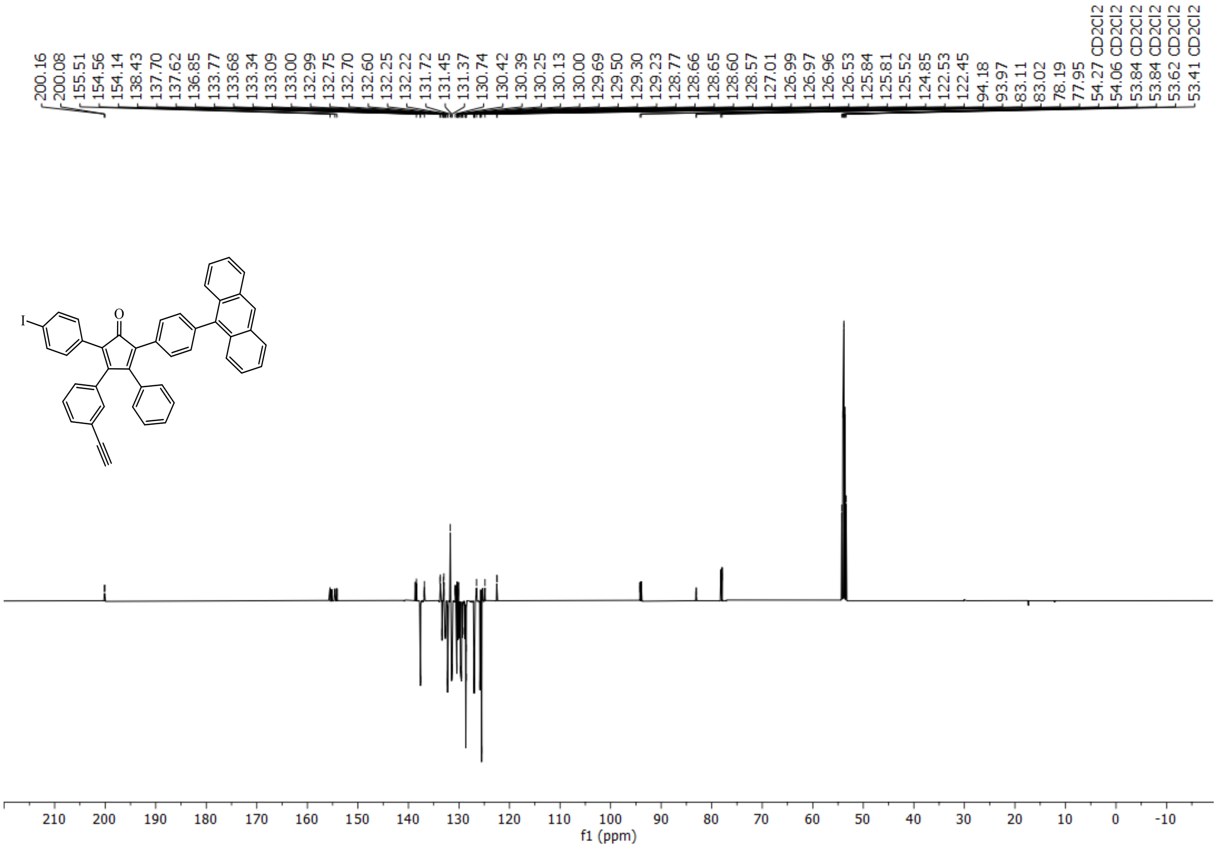

Figure S10. ^{13}C NMR spectrum of compound **5** (75 MHz, CD_2Cl_2, 298K).

Characterization of "clickable" GNRs applied as biointerface

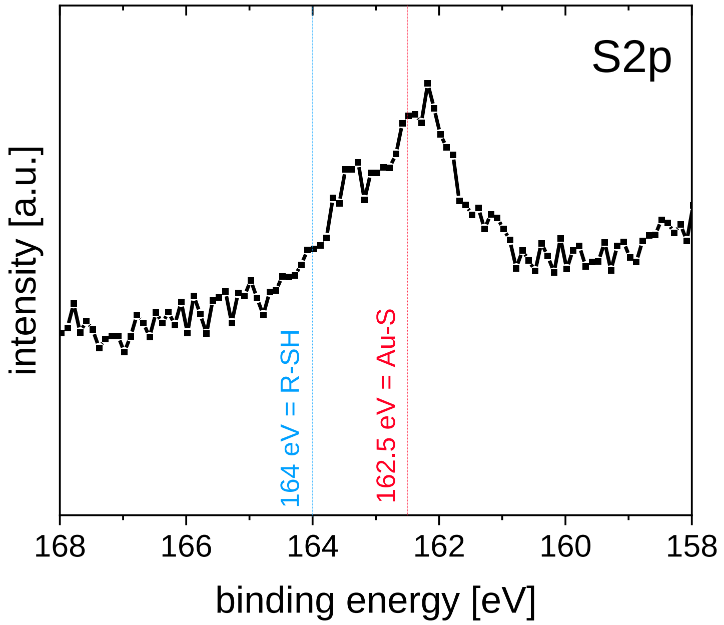

Figure S11. High-resolution XPS S2p signal of the SAM-coated gold working electrode.

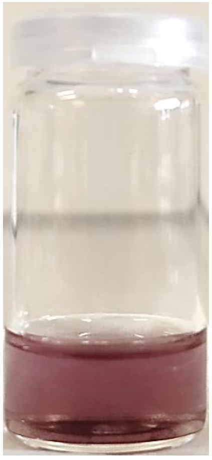

Figure S12. Representative photograph of the resulting **TIPS-GNR** (**10**) dispersion in THF after sonication.

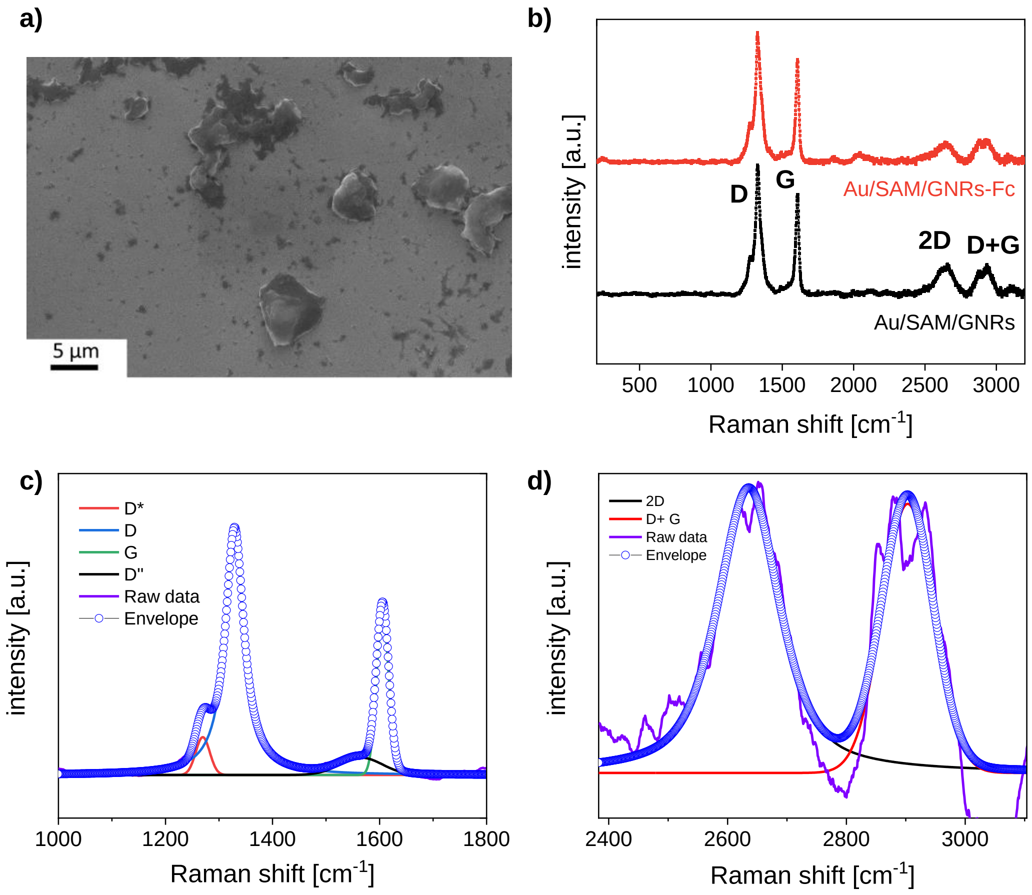

Figure S13. a) SEM image of GNR agglomerations on gold working electrode. b) Raman spectra of GNRs on gold working electrode before (black) and after (red) click-functionalization with N_3-Fc. Deconvolution of the c) D and G, and d) 2D and D+G bands of the Raman spectrum.

Calculation of the average defect distance (L_D):[1]

$$L_D^2(nm^2) = (1.8 \pm 0.5) \times 10^{-9}\, \lambda_L^4 \left(\frac{I_D}{I_G}\right)^{-1} \qquad \text{Eq. S1}$$

where λ_L is the laser wavelength (nm), I_D the intensity of the D band, and I_G the intensity of the G band.

Calculation of defect density (n_D):[1]

$$n_D(cm^{-2}) = \frac{(1.8 \pm 0.5) \times 10^{22}}{\lambda_L^4} \left(\frac{I_D}{I_G}\right) \qquad \text{Eq. S2}$$

Radial breathing-like mode (RBLM):

The wavenumber of the RBLM, ν_{RBLM}, is nearly independent of the edge structure and can be roughly correlated with the GNR width, w (Å), according to[2]

$$\nu_{RBLM} = \frac{3.222\ \text{cm}^{-1}}{w} \qquad \text{Eq. S3}$$

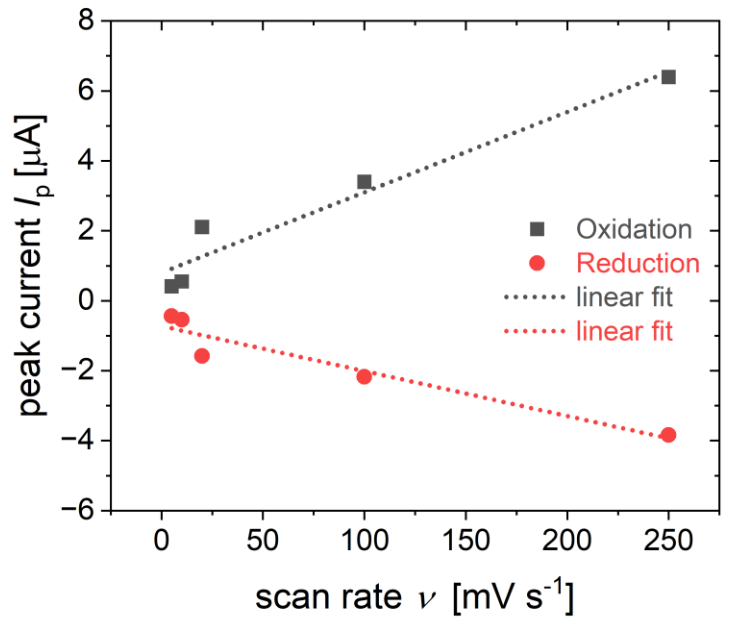

Figure S14. Peak current variation as function of the scan rate.

Electrochemical Impedance Spectroscopy (EIS) measurements were performed at different IL6 concentration to obtain more information about the electrochemical features of biosensing interface. As an example, Bode and Nyquist plots together with the obtained fitted curves are shown in **Figure S15a and b** for the IL6 concentration of 2.1 nM. The circuit used for the fitting is shown in **Figure S15c** and the results are summarized in **Table S1**.

The employed circuit was previously reported for the fitting of EIS measurements of different IL6 Au-aptamer electrochemical biosensors. [3,4] Briefly, the circuit consist of a solution resistance (R_s) connected in series to two parallel branches containing, in one side, a constant phase element (CPE), and in the other branch a series connection of the charge transfer resistance (R_{ct}) and a Warburg impedance (Z_w), which accounts for the diffusion of the redox couple.

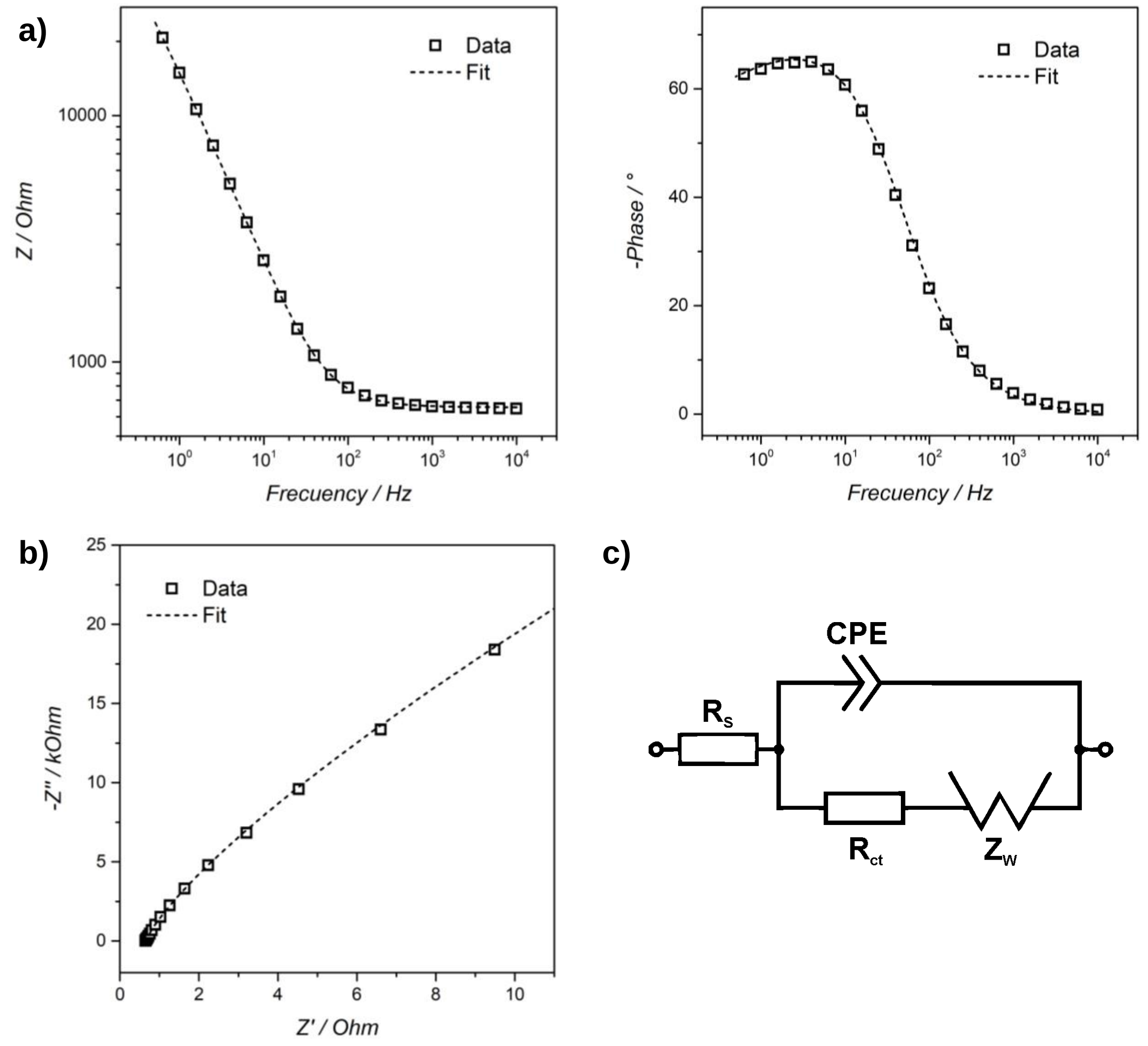

Figure S15. Bode (a) and Nyquist (b) plots with the respective fitting of the EIS spectrum for 2.1 nM IL6. Circuit employed for the fitting of the EIS spectra (c).

Table S1. Results for the fitting of the EIS spectra.

C_{IL6} [nM]	R_s [Ω]	R_{ct} [Ω]	CPE [S.s^n]	n_{CPE}	W [S.s^0.5]
**0.0**	744.35	-	7.39E-06	0.92496	2.95E-05
**0.2**	785.62	9.0549	7.70E-06	0.95968	2.26E-05
**0.4**	739.53	11.992	8.51E-06	0.93204	2.01E-05
**2.1**	655.36	53.933	7.51E-06	0.92325	1.27E-05
**4.2**	718.52	54.448	7.30E-06	0.9333	1.08E-05
**41.7**	756.05	10.523	5.47E-06	0.966	1.68E-05
**416.7**	775.04	10.112	5.64E-06	0.96689	1.68E-05
**2083.3**	774.14	-	4.27E-06	0.9935	2.00E-05

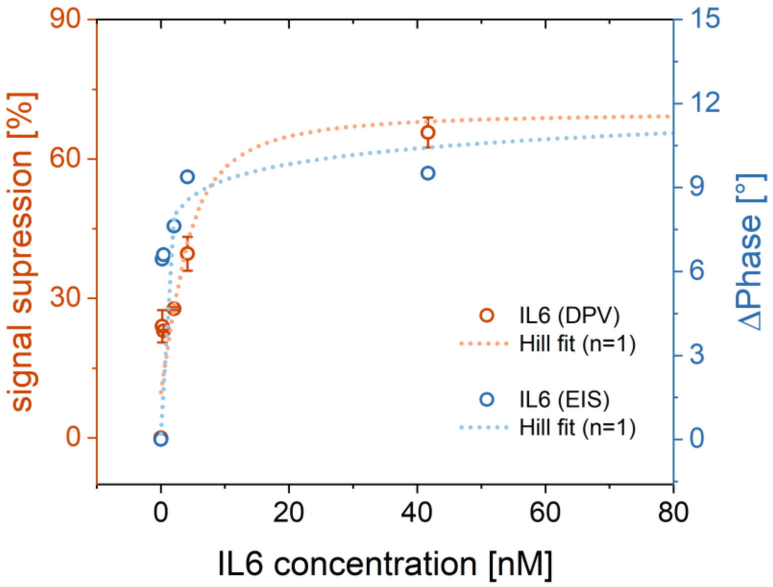

Figure S16. Magnification at low concentrations of the response curves obtained from DPV (red – signal suppression) and EIS (blue – phase angle shift) for the IL6 binding. Dashed lines indicate the fitting of the signals with the Hill model.

Table S2. Summary of the electrochemical IL6 sensor performance.

	**DPV**
sensitivity *S*	0.150 ± 0.006 % nM^{-1}
noise *σ*	0.165 %
limit of detection (LOD)	3.3 nM
limit of quantification (LOQ)	11 nM
response time	1 min

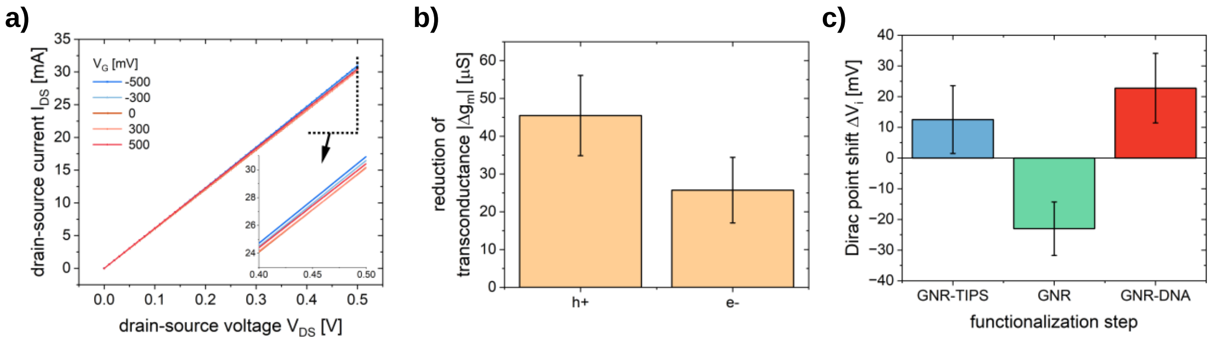

Figure S17. a) $I_{DS}V_{DS}$ output curves obtained at different applied V_G for GNR-functionalized rGO FETs. Change in transconductance of the hole (h^+) and electron (e^-) accumulation branch for the applied gate voltage range. c) Evaluated Dirac point shifts for the different functionalization steps of the rGO-based FETs (average of 3 devices).

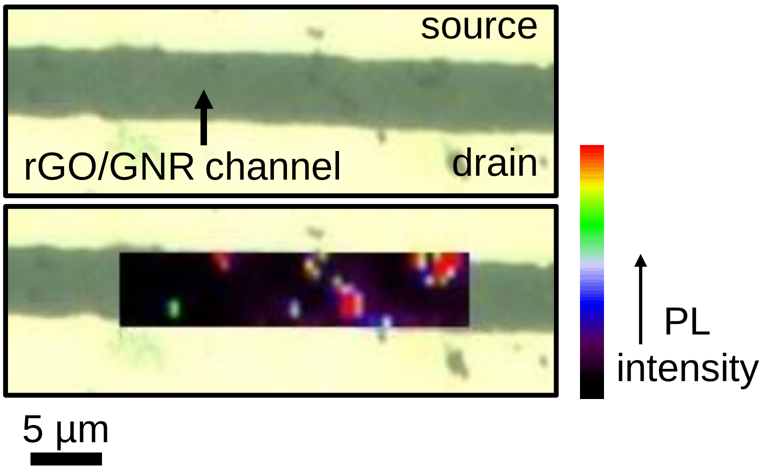

Figure S18. Image of the rGO/GNR transistor channel without (*top*) and with (*bottom*) overlay of the PL intensity mapping.

References

1 L. G. Cançado, A. Jorio, E. H. M. Ferreira, F. Stavale, C. A. Achete, R. B. Capaz, M. V. O. Moutinho, A. Lombardo, T. S. Kulmala and A. C. Ferrari, *Nano Lett.*, 2011, **11**, 3190–3196.

2 R. Gillen, M. Mohr and J. Maultzsch, *Phys. Rev. B*, 2010, **81**, 205426.

3 M. Tertis, P. I. Leva, D. Bogdan, M. Suciu, F. Graur and C. Cristea, *Biosensors and Bioelectronics*, 2019, **137**, 123–132.

4 M. Tertiş, B. Ciui, M. Suciu, R. Săndulescu and C. Cristea, *Electrochimica Acta*, 2017, **258**, 1208–1218.
